# Supplementary material for: Processing supramolecular framework for free interconvertible liquid separation
Source: Nat Commun. 2020 Jan 22;11:425. doi: 10.1038/s41467-019-14227-6 (PMC6976700; doi:10.1038/s41467-019-14227-6)
Supplement: Supplementary file 1 — Supplementary Information [file 41467_2019_14227_MOESM1_ESM.pdf]

Electronic Supplementary Information

Processing supramolecular framework for free interconvertible liquid separation

Zhang et al.

Supplementary Methods

Materials. The general chemicals, 1,4-dibromobutane, 1,6-dibromohexane, 1,8-dibromooctane, 1,10-dibromodecane, 1,4-dimethoxybenzene, paraformaldehyde, 5-bromopentanenitrile, adiponitrile (ADN), boron fluoride ethyl ether ([$BF_3 \cdot O(C_2H_5)_2$]) are the products of J&K Scientific Ltd. $H_4SiW_{12}O_{40}$ (SiW), trimethylamine (TEA), hydroquinone, 4-methoxyphenol and solvents were purchased from Sinopharm Chemical Reagent Co., Ltd. All the compounds were used without further purification. All the solvents were analytical grade and used as received except that 1,2-dichloroethane (DCE) was dried with activated molecular sieves (4A) for days and distilled just before using. $H_4PMo_{11}VO_{40}$ (PMoV) [1] and $K_4PW_{11}VO_{40}$ (PWV) [2] were freshly synthesized according to the previous literatures. Doubly distilled water was used in the experiments. Silica gel (300−400 mesh) was applied for column chromatography.

Measurements. ^{1}H NMR and 2D NOESY NMR spectra were recorded on a Bruker Avance 500 MHz spectrometer (Germany) by using tetramethylsilane (TMS) as internal reference (s= singlet, br= broad, d =doublet, t= triplet, q= quartet, m= multiplet). FT-IR spectra (KBr pellet) were collected on a Bruker Vertex 80 V spectrometer (Germany) equipped with DTGS detector (32 scans) in a resolution of 4 cm^{-1}. Organic elemental analyses (C, H, N) were carried out on a Vario micro cube from Elementar Germany. MALDI-TOF mass spectra were recorded on a matrix assisted laser desorption ionization (MALDI) time of flight (TOF) mass spectrometer (Bruker Autoflex^{TM} speed TOF/TOF, Germany), equipped with a nitrogen laser (337 nm, 3 ns pulse). The matrix was trans-2-[3-(4-tert-Butylphenyl)-2-methyl-2-propenylidene] malononitrile (DCTB). The mass to charge ratio range during datum acquisition is from 700 to 2000 Da for reflection positive mode and 5K to 20K Da for linear positive mode. GC mass spectra were carried out by ion trap gas chromatography (GC) mass spectrometer (Thermo Fisher-ITQ1100, USA) with electron impact (EI) ionization mode. Transmission electronic microscopic (TEM) images were obtained on a field emission electron microscope (JEOL JEM-2100F, Japan) with accelerating voltage of 200 KV without staining. Scanning electronic microscopic (SEM) measurement was performed on a JEOL JSM-6700F (Japan) field emission scanning electron microscope. Atomic force microscopic (AFM) images were taken with a Dimension FastScan^{TM} atomic force microscope (Bruker, USA) under ambient conditions. X-ray diffraction (XRD) data were recorded on a Rigaku SmartLab 3 (Japan) X-ray diffractometer using Cu Kα_1 radiation at wavelength of 1.542 Å. Static water contact angle (CA) measurements were performed with a Drop Shape Analysis System DSA10-MK2 (Krüss, Germany) at ambient temperature. Rheology measurements were executed on a Discovery Hybrid Rheometer (TA HR-1, USA). Isothermal titration calorimetric (ITC) data were obtained by using MicroCal VP-isothermal titration calorimeter (Malvern, UK). N_2 sorption experiment was carried out on a Quantachrome instruments ASiQMVH002-5 (USA). X-ray photoelectron spectra was carried out on an ESCALAB 250 spectrometer (Thermo, USA) with a monochromic X-ray source (Al Ka line, 1,486.6 eV) and the charging shift was corrected by the binding energy of C (1s) at 285.0 eV.

Supramolecular gel and fibrous assemblies. $(TBP)_4SiW$ (19.34 mg, 3 mmol) and BCB (1.63 mg, 6 mmol) at 2:1 molar ratio was mixed in 500 µL of chloroform. After 4 h sonication of the solution the supramolecular gel formed for structural measurements and post-treatment. The xerogel powder dissolves in dimethyl sulfoxide (DMSO), dimethyl formamide (DMF), formamide, and dichloromethane, but not in water, ethylene glycol, glycerol, methanol, ethanol, acetonitrile, acetone, tetrachloromethane, toluene, and hexane et. al. The long fibers were prepared by using the same procedure within a shorter sonication time, such as 1−3 h before the formation of gels. For other gel samples prepared from others supramolecular hosts, $(TBP)_4PWV$, $(TBP)_4PMoV$, $(THP)_4SiW$, $(TOP)_4SiW$, and $(TDP)_4SiW$ were used following the same methods under different concentrations.

Framework assembly gel membranes. The gel membranes were prepared through a simple spin-coating procedure. The chloroform solution for spin coating was prepared from diluting above prepared supramolecular gel or the pre-gelation solution after sonicating 3 h. Commercial solid substrates, stainless steel mesh, nonwoven, filter paper, silicon wafer, and copper grid, were used depending on the purpose for various measurements or separations. The spinning speeds were set at 400 r·s^{-1} for 30 s and then 2000 r·s^{-1} for 60 s. The concentration was normally 1.00 mM based on $(TBP)_4SiW$ (if not specified). After dryness,

the prepared membranes were applied for measurements. Here, some changed preparation conditions differing from that of the membrane for separation were made for getting a clear structural observation depending on the instruments.

Samples for TEM measurement. To obtain a higher resolution image of the gel nanoparticles, a dilute gel solution was used for TEM measurement, because concentration in gel state is difficult to get clear images. The chloroform solutions with concentrations of 0.25 mM, 0.05 mM, 0.01 mM, based on $(TBP)_4SiW$, was used. Here, we used a thin copper ring to capture a thin membrane and then cast onto a copper grid at once. During the measurement, high-energy electron beam was used to sweep the surface of assemblies in an instant repeatedly to get an image with clear contrast. Smart camera technique was used for the image collection. The position and size of clusters showed a slight distortion because of the ghosting phenomenon of image superposition.

For observation of the gelation versus sonication time, we fixed the concentration at 0.25 mM based on $(TBP)_4SiW$. During the observation for the formation of fibers versus the sonication, samples were taken every 1.0 h.

Samples for SEM measurement. The samples for SEM measurement were prepared by spin-coating gel solution onto different substrates. For top morphologic observation, the samples were spin-coated on the steel mesh with a concentration of 1.00 mM, based on $(TBP)_4SiW$ (1.5 mL). For the section images, the same sample was spin-coated onto a silicon wafer within the same area, and the thickness of gel membrane was controlled by changing coating volume.

Samples for rheology measurement. The gels used for rheology measurement were prepared at a concentration of 10 mM based on supramolecular hosts. The gap distance between two 25 mm ETC stainless parallel plates was 0.2 mm and all experiments were carried out at temperature of 25 °C. Oscillatory frequency sweeps were set in the range from 0.1 to 100 rad s^{-1} at an oscillation amplitude (g= 0.5%). The cyclic experiments were conducted under a continuous angular frequency (ω =10.0 rad s^{-1}) with alternating oscillation amplitude between 0.5% and 100%.

ITC titration experiment. TBP·Br and BCB were dissolved in chloroform at a concentration of 8.0 mM and 0.2 mM respectively. BCB (1.4 mL) was titrated by TBP·Br with a rate of 5.0 μL min^{-1}.

N_2 sorption experiment. The experiment was carried out at 77K on a Quantachrome instruments ASiQMVH002-5 after a pretreatment, in which samples were maintained under vacuum at 50 °C for 8.0 h. The pore size distribution and pore volume were estimated by the NLDFT (Non-local Density Functional Theory) method and BET (Brunauer–Emmett–Teller) method for the micropores and macropores. Independent experiment from different instrument was carried out to ensure the accuracy.

Samples for static CAs measurement. The sample on the steel mesh by a spin-coating method was prepared for CA measurement. The chloroform solution at a concentration of 1.00 mM for $(TBP)_4SiW$ (1.5 mL), prepared from diluting the above prepared supramolecular gel was spin-coated on a steel mesh. The value was an average value obtained by measuring five different positions.

Membrane devices, separation efficiency and flux calculation. For a simple separation device, an absorbent cotton ball was stuffed to the neck of a dropping pipette and covered with a 0.2 mm of xerogel powder. 500 μL of $CHCl_3$ was added to swell the xerogel for 10 min, and let the excess solvent pass through. The separation experiment was then carried out for the mixture of oil and water under gravity. To keep avoid any underfilling induced leakage, the xerogel powder which was obtained through evaporation at room temperature was grinded carefully.

For a flat substrate, the framework gel membrane coating on stainless steel mesh via spin-coating was nipped by two glass tubes bearing ground rims with an inner diameter of ca. 2.0 cm, in which one tube has a melt sand layer to support the stainless-steel mesh. The dyed oily liquids, water, and oil/water mixtures, were then poured onto the glass tube for separation under gravity. For organic liquids with relative density higher than water, a vertical device was used, while for light oily components, a tilting tube device was set so that the top organic liquid phases with relative density lower than water can touch and flow past the membrane.

To realize interconvertible separation, methanol, ethanol, isopropanol and tetrahydrofuran (because they are water miscible and do not damage the membrane) were used as the joystick liquid to modulate the surface wetting property. For the switching from oleophilic membrane (oily liquid passing through) to hydrophilic membrane (water permeation), the above prepared membrane was firstly wetted with the joystick liquid (for example methanol) and then water. Then, the membrane device was applied for the separation, in which water passes through but oil is blocked spontaneously. After the membrane was washed with joystick liquid methanol again and followingly oil liquids (for example carbon tetrachloride), as shown in Video 1, the membrane device was applied for the separation of mixture of oil and water, for which only oily liquids pass through the membrane spontaneously.

Separation efficiency (η) was determined by:

$$\eta = \frac{m}{m_0} \times 100\ \%$$

where m and m_0 are the weight of liquids to be separated before and after the separation. The possible adsorption difference was ignored, because pre-wetting to the membrane was carried out before the separation experiments. The last five parallel experiments were adopted to get average values.

Flux (J) under gravity was calculated by:

$$J = \frac{V}{t \times A}$$

where V is the filtrate volume (in L), A is the effective filtration membrane area (in m^2), and t is the separation time (h). The data were collected from the average of five parallel experiments.

Synthesis of ionic complexes and ditopic guest

Cationic pillar[5]arene derivatives. The synthesis routes shown in the following route.

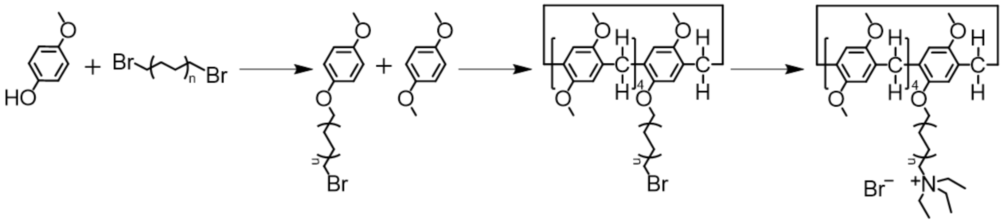

n=1, BBMB
n=2, BHMB
n=3, BOMB
n=4, BDMB

n=1, BBP
n=2, BHP
n=3, BOP
n=4, BDP

n=1, TBP·Br
n=2, THP·Br
n=3, TOP·Br
n=4, TDP·Br

1-(4'-Bromobutoxy)-4-methoxy-benzene (BBMB). To a stirred solution of 1,4-dibromobutane (12.94 g, 60 mmol) in acetone (300 mL) was added K_2CO_3 (8.28 g, 60 mmol) and 4-methoxyphenol (1.24 g, 10 mmol). The mixture was stirred and refluxed overnight. After the reaction was completed, the solid residue was removed by filtration and the filtrate was evaporated under reduced pressure. Further purification was carried out via column chromatography on silica gel with CH_2Cl_2/n-hexane (2:3, in v/v) as eluent, giving 2.48 g of product. Yield: 95.7%. ^{1}H NMR ($CDCl_3$, 500 MHz, 25°C): δ (ppm)= 6.83 (s, 4H), 3.96−3.93 (t, 2H), 3.77 (s, 3H), 3.50−3.47 (t, 2H), 2.09−2.04 (m, 2H), 1.95−1.89 (m, 2H), as shown in Supplementary Figure 1.

4-Bromobutyloxy pillar[5]arene (BBP). BBP was synthesized with an appropriate modification to a published method [3]. BBMB (1.44 g, 5.54 mmol), 1,4-dimethoxybenzene (3.06 g, 22.16 mmol), and paraformaldehyde (0.84 g, 27.70 mmol) were added into anhydrous 1,2-dichloroethane (100 mL). The reaction solution was stirred under N_2 atmosphere for 10 min, and then boron fluoride ethyl ether ([$BF_3 \cdot O(C_2H_5)_2$]) (3.46 mL, 27.70 mmol) was added. The mixture was further stirred at 28°C for 3.5 h. A mixture of water and methanol (1:1 in v/v, 100 mL) was added and stirring overnight. The solution was extracted with CH_2Cl_2,

the insoluble solid was filtered out, and then, the filtrate was dried over Na_2SO_4. After evaporating excess solvent under reduced pressure, further purification was carried out by column chromatography on silica gel with CH_2Cl_2/n-hexane (2:1 in v/v) as eluent, giving 1.55 g of product. Yield: 32%. ^{1}H NMR ($CDCl_3$, 500 MHz, 25°C): δ (ppm)= 6.82−6.74 (m, 10H), 3.88−3.86 (t, 2H), 3.82−3.77 (m, 10H), 3.72−3.64 (m, 27H), 3.37−3.35 (t, 2H), 1.97 (m, 2H), 1.85 (m, 2H), as shown in Supplementary Figure 2. MALDI-TOF-MS (m/z) $[M+H]^+$: calculated for $C_{48}H_{55}O_{10}BrH$: 872.856, found: 872.897, as shown in Supplementary Figure 3. Elemental analysis for $C_{48}H_{55}O_{10}Br$ (871.85 g/mol): C, 66.13%; H, 6.36%, found: C, 66.34%; H, 6.15%.

4-triethylammonium butoxylpillar[5]arene bromide (TBP·Br). BBP (1.00 g, 1.15 mmol) and triethylamine (1.16 g, 11.5 mmol) were mixed in acetonitrile (50 mL), and the solution was stirred and refluxed for 48 h. After cooled to room temperature, saturated brine solution was added to the reaction solution. The mixture was extracted with chloroform (20 mL×3), and dried over anhydrous Na_2SO_4. After evaporating excess solvent under reduced pressure, further purification of the residue was carried out by column chromatography on silica gel with CH_2Cl_2/methanol (100:1 to 20:1 in v/v) as eluent, giving 0.82 g of product. Yield: 73.3%. ^{1}H NMR ($CDCl_3$, 500 MHz, 25°C): δ (ppm)= 6.81−6.52 (m, 10H), 3.97−3.93 (t, 2H), 3.80−3.72 (m, 10H), 3.69−3.58 (m, 24H), 3.48−3.44 (t, 2H), 3.44−3.42 (s, 3H), 3.37−3.29 (q, 6H), 1.99−1.88 (m, 4H), 1.25−1.16 (t, 9H), as shown in Supplementary Figure 4. ^{13}C NMR ($CDCl_3$, 500 MHz, 25°C): δ (ppm)= 151.26, 151.15, 151.11, 151.02, 150.93, 150.87, 150.82, 150.76, 149.46, 129.07, 129.01, 128.74, 128.65, 128.50, 128.46, 128.25, 128.21, 128.06, 127.93, 115.17, 115.02, 114.87, 114.47, 114.43, 114.40, 114.17, 114.08, 114.01, 67.23, 57.39, 56.61, 56.39, 56.33, 55.96, 55.88, 55.82, 55.71, 30.27, 30.24, 29.72, 29.52, 29.43, 26.42, 19.37, 7.87, as shown in Supplementary Figure 5. MALDI-TOF-MS (m/z) $[M]^+$: calculated for $C_{54}H_{70}O_{10}N$: 893.134, found: 892.765, as shown in Supplementary Figure 6. Elemental analysis for $C_{54}H_{70}O_{10}NBr$ (973.038 g/mol): C, 66.66%; H, 7.25%; N, 1.44%, found: C, 66.28%; H, 7.29%; N, 1.40%.

4-triethylammonium hexyloxylpillar[5]arene bromide (THP·Br). THP·Br was synthesized under the same conditions as the synthesis of TBP·Br but using 1,6-dibromohexane as the initial reactant. Yield: 78.9%. ^{1}H NMR ($CDCl_3$, 500 MHz, 25°C): δ (ppm)= 6.79−6.66 (m, 10H), 3.90−3.84 (t, 2H), 3.79−3.72 (m, 10H), 3.68−3.55 (m, 27H), 3.50−3.42 (q, 6H), 3.28−3.19 (t, 2H), 1.82−1.74 (m, 2H), 1.68−1.60 (m, 2H), 1.55−1.47 (m, 2H), 1.41−1.31 (m, 11H), as shown in Supplementary Figure 7. ^{13}C NMR($CDCl_3$, 500 MHz, 25°C): δ (ppm)= 151.03, 150.95, 150.92, 150.90, 150.89, 150.86, 150.78, 149.93, 128.60, 128.41, 128.38, 128.33, 128.29, 128.25, 128.18, 128.07, 115.08, 114.48, 114.45, 114.34, 114.28, 114.24, 114.09, 114.06, 114.02, 68.03, 57.64, 56.02, 55.98, 55.89, 55.84, 55.73, 29.95, 29.74, 29.69, 29.52, 29.50, 26.21, 25.97, 22.15, 8.09, as shown in Supplementary Figure 8. MALDI-TOF-MS (m/z) $[M]^+$: calculated for $C_{56}H_{74}O_{10}N$: 921.188, found: 921.274, as shown in Supplementary Figure 9. Elemental analysis for $C_{56}H_{74}O_{10}NBr$(1001.09 g/mol): C, 67.19%; H, 7.45%; N, 1.40%, found: C, 67.05%; H, 7.39%; N, 1.38%.

4-triethylammonium octyloxylpillar[5]arene bromide (TOP·Br). This product was synthesized via the same procedures as that of synthesis of TBP·Br but using 1,8-dibromooctane as the initial reactant. Yield: 75.6%. ^{1}H NMR ($CDCl_3$, 500 MHz, 25°C): δ (ppm)= 6.82−6.71 (m, 10H), 3.91−3.84 (t, 2H), 3.80−3.72 (m, 10H), 3.68−3.59 (m, 27H), 3.51−3.43 (q, 6H), 3.18−3.11 (t, 2H), 1.80−1.71 (m, 2H), 1.51−1.42 (m, 4H), 1.42−1.35 (t, 9H), 1.31−1.23 (m, 2H), 1.18−1.04 (m, 4H), as shown in Supplementary Figure 10. ^{13}C NMR($CDCl_3$, 500 MHz, 25°C): δ (ppm)= 150.95, 150.90, 150.89, 150.86, 150.85, 150.81, 150.79, 150.72, 150.04, 128.43, 128.39, 128.33, 128.28, 128.25, 128.24, 128.20, 114.99, 114.57, 114.50, 114.47, 114.30, 114.27, 114.14,114.12, 114.02, 68.27, 57.75, 56.11, 56.10, 56.09, 56.00, 55.92, 55.86, 55.83, 55.82, 55.78, 29.85, 29.76, 29.63, 29.42, 29.40, 29.04, 28.82, 26.14, 25.86, 22.10, 8.13, as shown in Supplementary Figure 11. MALDI-TOF-MS (m/z) $[M]^+$: calculated for $C_{58}H_{78}O_{10}N$: 949.241, found: 949.405, as shown in Supplementary Figure 12. Elemental analysis for $C_{58}H_{78}O_{10}NBr$ (1029.15 g/mol): C, 67.69%; H, 7.64%; N, 1.36%, found: C, 67.72%; H, 7.54%; N, 1.35%.

4-triethylammonium decyloxylpillar[5]arene bromide (TDP·Br). TDP·Br was synthesized with the same procedures as the synthesis of TBP·Br but using 1,10-dibromodecane as the initial reactant. Yield: 82.7%. ^{1}H NMR ($CDCl_3$, 500 MHz, 25°C): δ (ppm)= 6.85−6.73 (m, 10H), 3.93−3.86 (t, 2H), 3.79−3.72 (m, 10H), 3.71−3.58 (m, 27H), 3.53−3.45 (q, 6H), 3.07−3.00 (t, 2H), 1.81−1.72 (m, 2H), 1.55−1.49 (m, 2H), 1.44−1.37 (t, 9H), 1.36−1.31 (m, 2H), 1.31−1.24 (m, 2H), 1.24−1.14 (m, 2H), 0.99−0.89

(m, 2H), 0.76−0.61 (m, 4H), as shown in Supplementary Figure 13. ^{13}C NMR ($CDCl_3$, 500 MHz, 25°C): δ (ppm)=150.96, 150.88, 150.87, 150.79, 150.78, 150.75, 150.63, 150.13, 128.59, 128.50, 128.40, 128.38, 128.31, 128.26, 128.24, 114.79, 114.65, 114.56, 114.53, 114.04, 113.99, 113.94, 113.91, 68.36, 56.28, 56.26, 56.21, 56.17, 56.15, 55.79, 55.77, 55.71, 29.79, 29.62, 29.58, 29.37, 29.32, 29.05, 29.03, 29.01, 28.88, 26.22, 25.92, 21.97, 8.08, as shown in Supplementary Figure 14. MALDI-TOF-MS (m/z) $[M]^+$: calculated for $C_{60}H_{82}O_{10}N$: 977.294, found: 977.444, as shown in Supplementary Figure 15. Elemental analysis for $C_{60}H_{82}O_{10}NBr$ (1057.20 g/mol): C, 68.17%; H, 7.82%; N, 1.33%, found: C, 68.24%; H, 7.88%; N, 1.37%.

Ionic complex (TBP)$_4$SiW. To a solution of TBP·Br (1.00 g, 1.03 mmol) in methanol (20 mL), $H_4SiW_{12}O_{40}$ (SiW, 719.54 mg, 0.25 mmol) in methanol (20 mL) was added slowly with vigorous stirring at room temperature. After 2 h of ionic exchange reaction, the formed precipitate was filtered and washed with deionized water (30 mL×3) and then methanol (30 mL×3), dried under vacuum, giving ionic complex (TBP)$_4$SiW (1.41 g). Yield: 87.5%. ^1H NMR ($CDCl_3$, 500 MHz, 25°C): δ (ppm)= 6.83−6.56 (br, 10H), 3.94−3.86 (br, 2H), 3.80−3.70 (br, 10H), 3.69−3.48 (br, 27H), 3.44−3.36 (br, 6H), 3.35−3.28 (br, 2H), 2.05−1.89 (br, 4H), 1.46−1.29 (br, 9H), as shown in Supplementary Figure 16. MALDI-TOF-MS (m/z) $[M+TBP]^+$: calculated for $(C_{54}H_{70}O_{10}N)_5SiW_{12}O_{40}$: 7739.813, found: 7739.574, as shown in Supplementary Figure 17. FT-IR (KBr pellet): *v*= 3016−2805, 1505, 1464, 1400, 1344−1285, 1252, 1211, 1178, 1099, 1051, 1045, 1011, 969, 923, 883, 803, 531 cm^{-1}, as shown in Supplementary Figure 18. Elemental analysis for $(C_{54}H_{70}O_{10}N)_4SiW_{12}O_{40}$ (6446.68 g/mol): C, 40.24%; H, 4.38%; N, 0.87%, found: C, 40.27%; H, 4.36%; N, 0.87%.

Ionic complex (TBP)$_4$PWV. To a solution of TBP·Br (1.00 g, 1.03 mmol) in methanol/water (3:2, 20 mL), $K_4PW_{11}VO_{40}$ (PWV, 725.13 mg, 0.25 mmol) in methanol/water (3:2, 20 mL) was added slowly with vigorous stirring at 50°C. After 2 h of ionic exchange reaction, the formed precipitate was filtered and washed with deionized water (50°C, 30 mL×3) and then methanol (30 mL×3), dried under vacuum, giving ionic complex (TBP)$_4$PWV (1.30 g). Yield: 82.3%. ^1H NMR ($CDCl_3$, 500 MHz, 25°C): δ (ppm)= 6.83−6.51 (br, 10H), 3.95−3.85 (br, 2H), 3.80−3.70 (br, 10H), 3.68−3.45 (br, 27H), 3.44−3.34 (br, 6H), 3.33−3.23 (br, 2H), 2.04−1.86 (br, 4H), 1.47−1.29 (br, 9H), as shown in Supplementary Figure 19. MALDI-TOF-MS (m/z) $[M+TBP]^+$: calculated for $(C_{54}H_{70}O_{10}N)_5PW_{11}VO_{40}$: 7209.803, found: 7209.686, as shown in Supplementary Figure 20. FT-IR (KBr pellet): *v*= 3026−2791, 1502, 1465, 1399, 1345−1283, 1253, 1214, 1176, 1096, 1071, 1045, 968, 929, 890, 883, 814, 521 cm^{-1}, as shown in Supplementary Figure 21. Elemental analysis for $(C_{54}H_{70}O_{10}N)_4PW_{11}VO_{40}$ (6316.67 g/mol): C, 41.07%; H, 4.47%; N, 0.89%, found: C, 41.12%; H, 4.52%; N, 0.87%.

Ionic complex (TBP)$_4$PMoV. To a solution of TBP·Br (1.00 g, 1.03 mmol) in methanol (20 mL), $H_4PMo_{11}VO_{40}$ (PMoV, 445.32 mg, 0.25 mmol) in methanol (20 mL) was added slowly with vigorous stirring at room temperature. After 2 h of ionic exchange reaction, the formed precipitate was filtered and washed with deionized water (30 mL×3) and then methanol (30 mL×3), dried under vacuum, giving ionic complex (TBP)$_4$PMoV (1.08 g). Yield: 80.7%. ^1H NMR ($CDCl_3$, 500 MHz, 25°C): δ (ppm)= 6.81−6.51 (br, 10H), 3.94−3.82 (br, 2H), 3.80−3.70 (br, 10H), 3.70−3.44 (br, 27H), 3.43−3.33 (br, 6H), 3.33−3.21 (br, 2H), 2.06−1.82 (br, 4H), 1.45−1.18 (br, 9H), as shown in Supplementary Figure 22. MALDI-TOF-MS (m/z) $[M+TBP]^+$: calculated for $(C_{54}H_{70}O_{10}N)_5PMo_{11}VO_{40}$: 6242.903, found: 6242.621, as shown in Supplementary Figure 23. FT-IR (KBr pellet): *v*= 3021−2800, 1503, 1464, 1400, 1343−1289, 1250, 1212, 1175, 1097, 1075, 1044, 986, 947, 875, 805, 501 cm^{-1}, as shown in Supplementary Figure 24. Elemental analysis for $(C_{54}H_{70}O_{10}N)_4PMo_{11}VO_{40}$ (5349.77 g/mol): C, 48.49%; H, 5.28%; N, 1.05%, found: C, 48.18%; H, 5.32%; N, 1.06%.

Ionic complex (THP)$_4$SiW. To a solution of THP·Br (1.00 g, 1.00 mmol) in methanol (20 mL), $H_4SiW_{12}O_{40}$ (SiW, 690.76 mg, 0.24 mmol) in methanol (20 mL) was added slowly with vigorous stirring at room temperature. After 2 h of ionic exchange reaction, the formed precipitate was filtered and washed with deionized water (30 mL×3) and then methanol (30 mL×3), dried under vacuum, giving ionic complex (THP)$_4$SiW (1.39 mg). Yield: 88.2%. ^1H NMR ($CDCl_3$, 500 MHz, 25°C): δ (ppm)= 6.85−6.63 (br, 10H), 3.92−3.84 (br, 2H), 3.83−3.72 (br, 10H), 3.70−3.55 (m, 27H), 3.55−3.41 (br, 6H), 3.20−3.05 (br, 2H), 1.91−1.79 (br, 2H), 1.78−1.68 (br, 2H), 1.61−1.58 (br, 2H), 1.52−1.38 (br, 11H), as shown in Supplementary Figure 25. MALDI-TOF-MS (m/z) $[M+THP]^+$: calculated for $(C_{56}H_{74}O_{10}N)_5SiW_{12}O_{40}$: 7480.079, found: 7480.310, as shown in Supplementary Figure 26. FT-IR

(KBr pellet): v= 3018−2797, 1502, 1465, 1401, 1343−1285, 1249, 1212, 1175, 1098, 1044, 1011, 971, 924, 883, 804, 534 cm^{-1}, as shown in Supplementary Figure 27. Elemental analysis for $(C_{56}H_{74}O_{10}N)_4SiW_{12}O_{40}$ (6558.89 g/mol): C, 41.02%; H, 4.55%; N, 0.85%, found: C, 41.41%; H, 4.51%; N, 0.81%.

Ionic complex host (TOP)$_4$SiW. To a solution of TOP·Br (1.00 g, 0.97 mmol) in methanol (20 mL), $H_4SiW_{12}O_{40}$ (SiW, 682.11 mg, 0.23 mmol) in methanol (20 mL) was added slowly with vigorous stirring at room temperature. After 2 h of ionic exchange reaction, the formed precipitate was filtered and washed with deionized water (30 mL×3) and then methanol (30 mL×3), dried under vacuum, giving ionic complex (TOP)$_4$SiW (1.34 g). Yield: 87.3%. ^{1}H NMR ($CDCl_3$, 500 MHz, 25°C): δ (ppm)= 6.80−6.68 (br, 10H), 3.86−3.80 (br, 2H), 3.79−3.70 (br, 10H), 3.69−3.55 (br, 27H), 3.52−3.38 (br, 6H), 3.12−3.00 (br, 2H), 1.84−1.74 (br, 2H), 1.72−1.61 (br, 2H), 1.53−1.48 (br, 2H), 1.48−1.39 (br, 9H), 1.39−1.27 (br, 6H), as shown in Supplementary Figure 28. MALDI-TOF-MS (m/z) $[M+TOP]^+$: calculated for $(C_{58}H_{78}O_{10}N)_5SiW_{12}O_{40}$: 7620.345, found: 7620.122, as shown in Supplementary Figure 29. FT-IR (KBr pellet): v= 3018−2797, 1502, 1465, 1401, 1343−1285, 1249, 1212, 1175, 1098, 1044, 1011, 971, 924, 883, 804, 534 cm^{-1}, as shown in Supplementary Figure 30. Elemental analysis for $(C_{58}H_{78}O_{10}N)_4SiW_{12}O_{40}$ (6671.10 g/mol): C, 41.77%; H, 4.71%; N, 0.84%, found: C, 41.36%; H, 4.63%; N, 0.80%.

Ionic complex (TDP)$_4$SiW. To a solution of TDP·Br (1.00 g, 0.95 mmol) in methanol (20 mL), $H_4SiW_{12}O_{40}$ (SiW, 664.01mg, 0.23 mmol) in methanol (20 mL) was added slowly with vigorous stirring at room temperature. After 2 h of ionic exchange reaction, the formed precipitate was filtered and washed with deionized water (30 mL×3) and then methanol (30 mL×3), dried under vacuum, giving ionic complex (TDP)$_4$SiW (1.29 g). Yield: 82.9%. ^{1}H NMR ($CDCl_3$, 500 MHz, 25°C): δ (ppm)= 6.86−6.68 (br, 10H), 3.85−3.80 (br, 2H), 3.79−3.72 (br, 10H), 3.70−3.57 (br, 27H), 3.53−3.43 (br, 6H), 3.30−3.13 (br, 2H), 1.81−1.71 (br, 2H), 1.60−1.55 (br, 2H) 1.54−1.41 (br, 11H), 1.38−1.30 (br, 2H), 1.30−1.23 (br, 2H), 1.23−1.08 (br, 6H), as shown in Supplementary Figure 31. MALDI-TOF-MS (m/z) $[M+TDP]^+$: calculated for $(C_{60}H_{82}O_{10}N)_5SiW_{12}O_{40}$: 7760.611, found: 7760.640, as shown in Supplementary Figure 32. FT-IR (KBr pellet): v= 3022−2788, 1504, 1464, 1400, 1342−1285, 1285, 1250, 1212, 1177, 1097, 1045, 1009, 969, 922, 882, 804, 531cm^{-1}, as shown in Supplementary Figure 33. Elemental analysis for $(C_{60}H_{82}O_{10}N)_4SiW_{12}O_{40}$ (6783.32 g/mol): C, 42.50%; H, 4.87%; N, 0.83%, found: C, 42.49; H, 4.85%; N, 0.84%. The XPS spectral measurements show the unchanged structure of clusters in all prepared ionic complexes, as summarized in Supplementary Figure 34.

para-Bis(4-cyanobutoxyl) benzene (BCB). Hydroquinone (1.10 g, 10 mmol), K_2CO_3 (8.29 g, 60 mmol) and 5-bromopentanenitrile (3.56 g, 22 mmol) were added into dried acetone (50 mL) and then the mixture solution was heated to reflux overnight. After the reaction cooling down, the precipitate was filtered, and the filtrate was evaporated under reduced pressure to remove excess solvent. The residue was purified via column chromatography on a silica gel with CH_2Cl_2 as eluent, giving white solid product of 1.52 g. Yield: 55.8%. ^{1}H NMR ($CDCl_3$, 500 MHz, 25°C): δ (ppm)= 6.84 (s, 4H), 4.00−3.95 (t, 4H), 2.49−2.45 (t, 4H), and 1.99−1.87 (m, 8H), as shown in Supplementary Figure 35. ^{13}C NMR ($CDCl_3$, 500 MHz, 25°C): δ (ppm)= 152.85, 119.35, 115.42, 67.28, 28.28, 22.48, 17.03, as shown in Supplementary Figure 36. GC-MS (m/z) $[M]^+$: calculated for $C_{16}H_{20}N_2O_2$: 272.34, found: 272.01, as shown in Supplementary Figure 37. Elemental analysis for $C_{16}H_{20}N_2O_2$ (272.34 g/mol): C, 70.56%; H, 7.40%; N, 10.29%, found: C, 70.89%; H, 7.26%; N, 10.25%.

Supplementary Figures

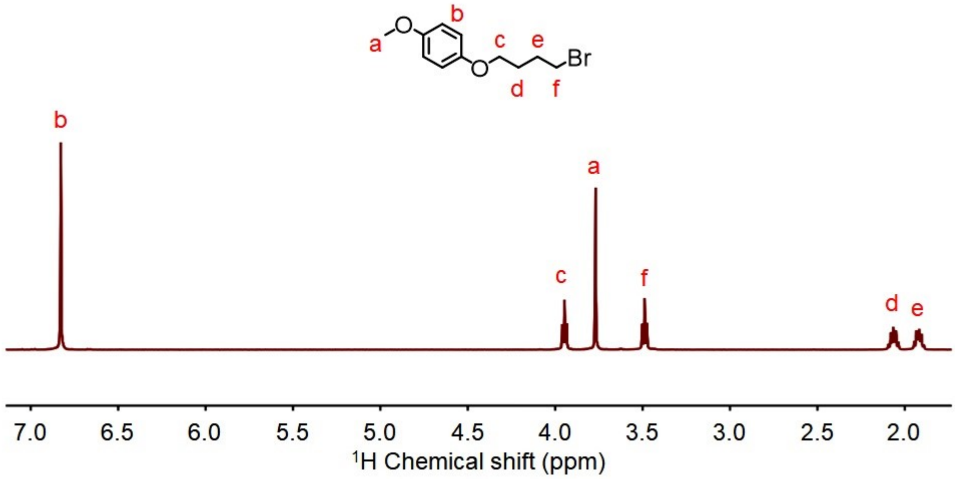

Supplementary Figure 1: ^{1}H NMR spectrum. BBMB in $CDCl_3$ (500 MHz, 25°C). Source data are provided as a Source Data file.

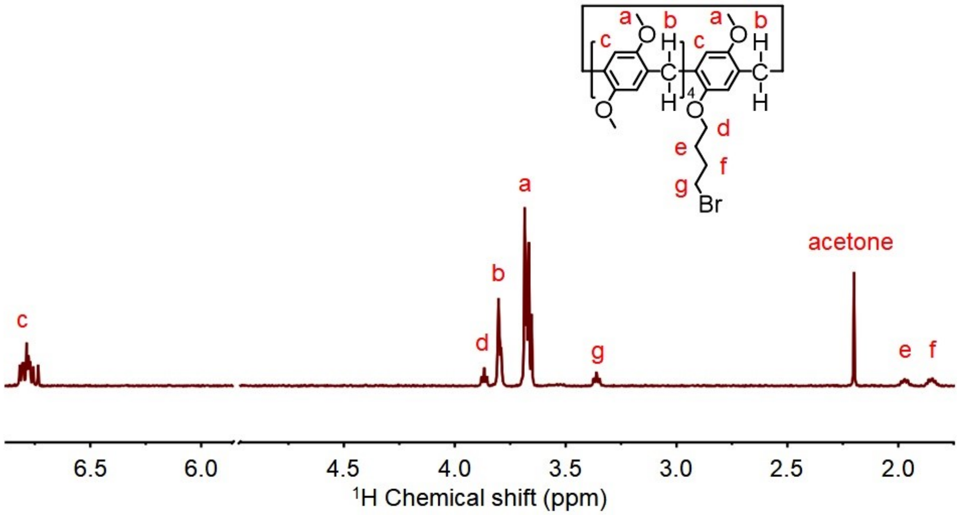

Supplementary Figure 2: ^{1}H NMR spectrum. BBP in $CDCl_3$ (500 MHz, 25°C). Source data are provided as a Source Data file.

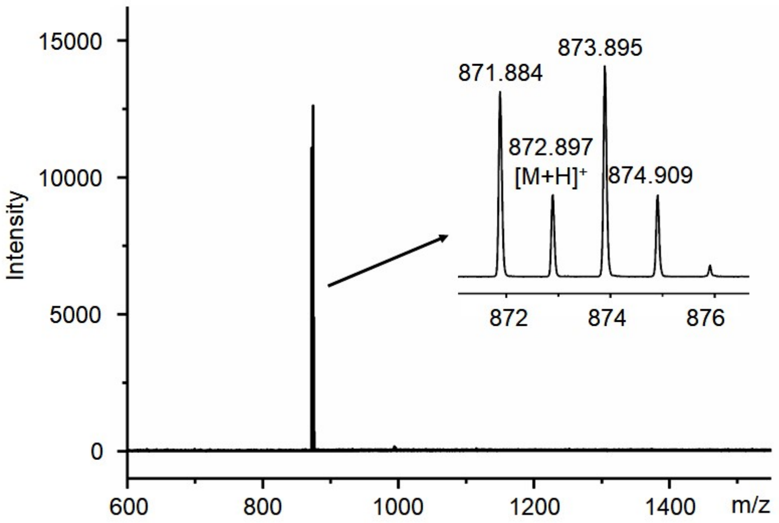

Supplementary Figure 3: MALDI-TOF MS. BBP. Source data are provided as a Source Data file.

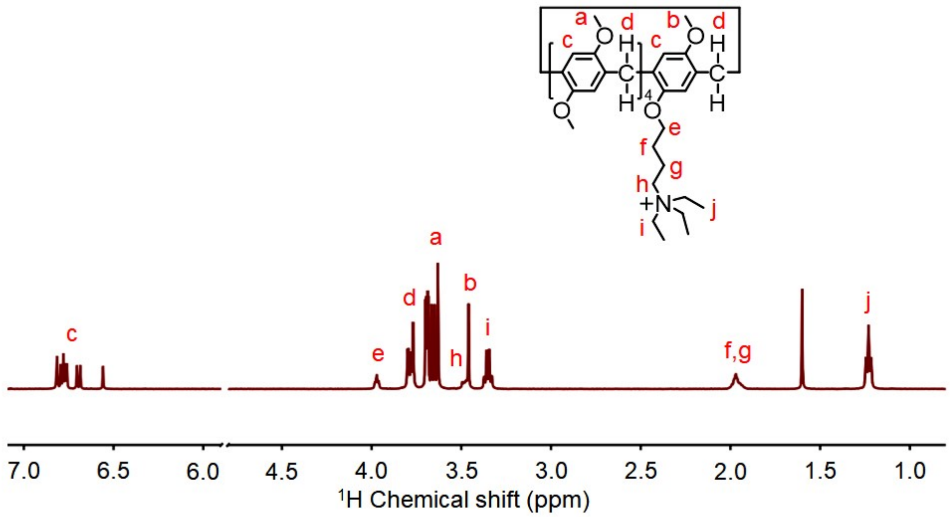

Supplementary Figure 4: ^{1}H NMR spectrum. TBP·Br in $CDCl_3$ (500 MHz, 25°C). Source data are provided as a Source Data file.

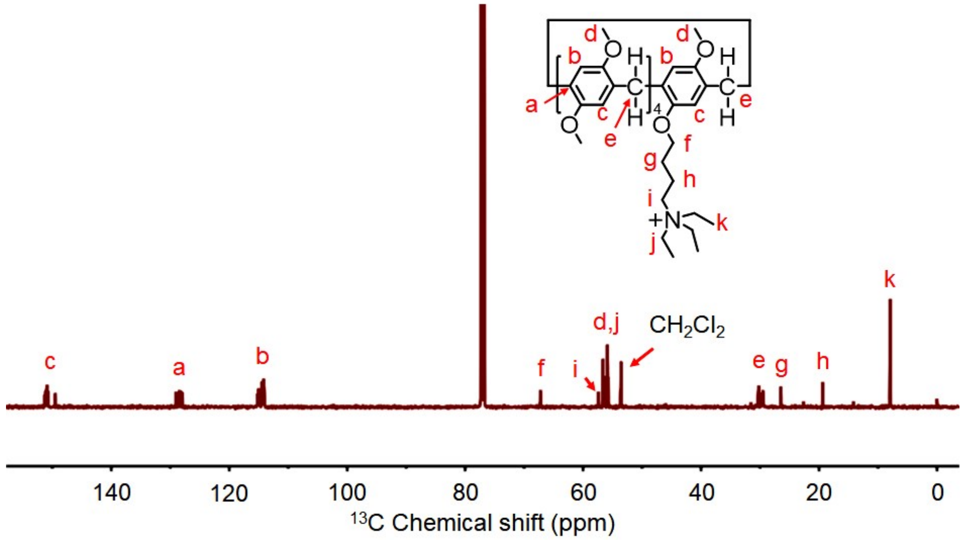

Supplementary Figure 5: ^{13}C NMR spectrum. TBP·Br in $CDCl_3$ (500 MHz, 25°C). Source data are provided as a Source Data file.

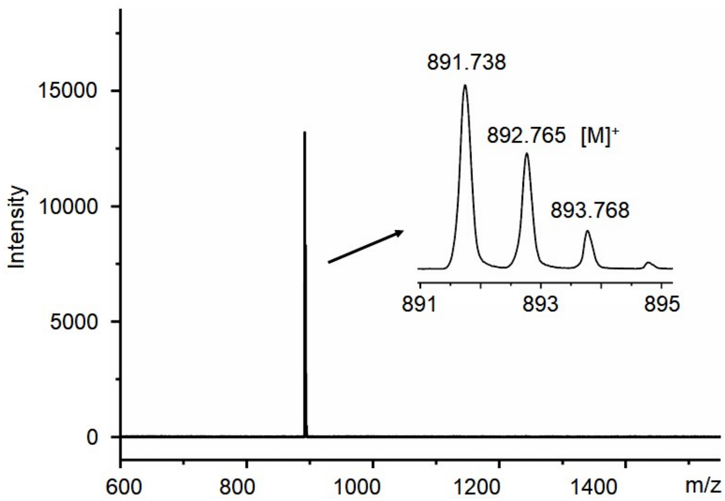

Supplementary Figure 6: MALDI-TOF MS. TBP·Br. Source data are provided as a Source Data file.

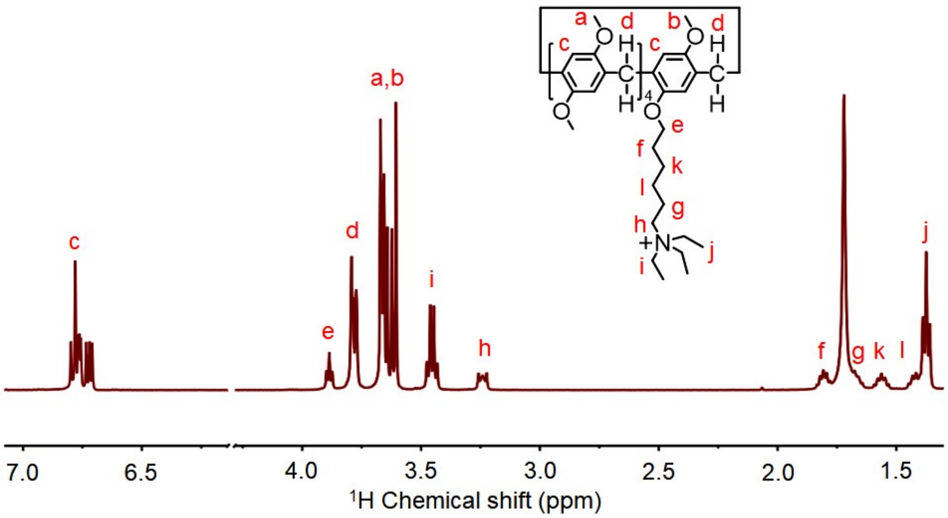

Supplementary Figure 7: ^{1}H NMR spectrum. THP·Br in $CDCl_3$ (500 MHz, 25°C). Source data are provided as a Source Data file.

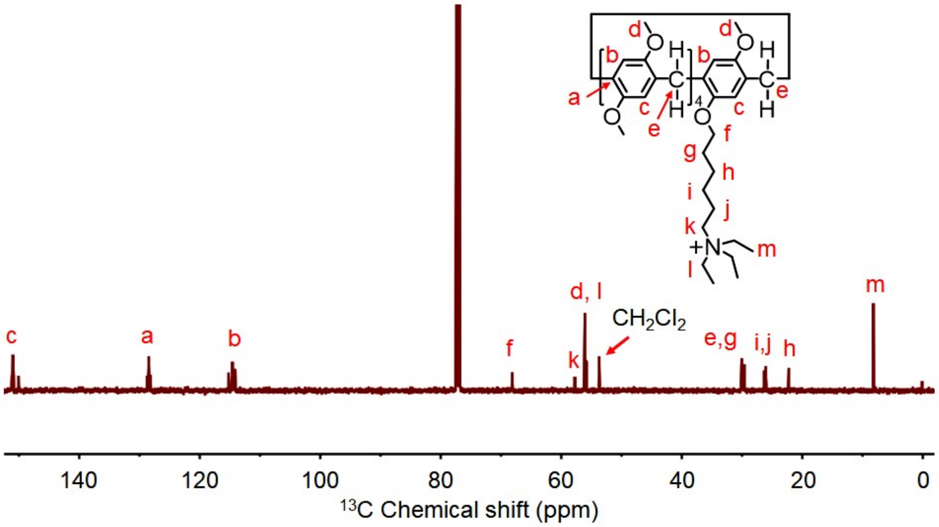

Supplementary Figure 8: ^{13}C NMR spectrum. THP·Br in $CDCl_3$ (500 MHz, 25°C). Source data are provided as a Source Data file.

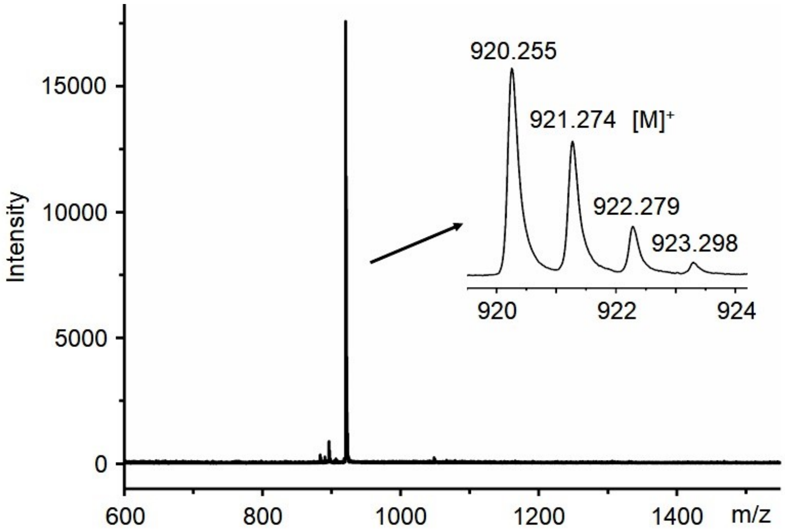

Supplementary Figure 9: MALDI-TOF MS. THP·Br. Source data are provided as a Source Data file.

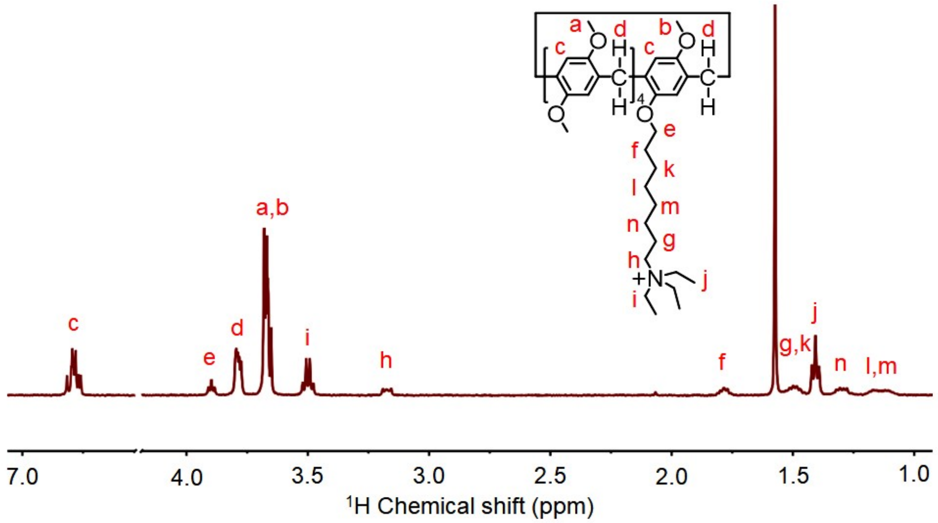

Supplementary Figure 10: ^{1}H NMR spectrum. TOP·Br in $CDCl_3$ (500 MHz, 25°C). Source data are provided as a Source Data file.

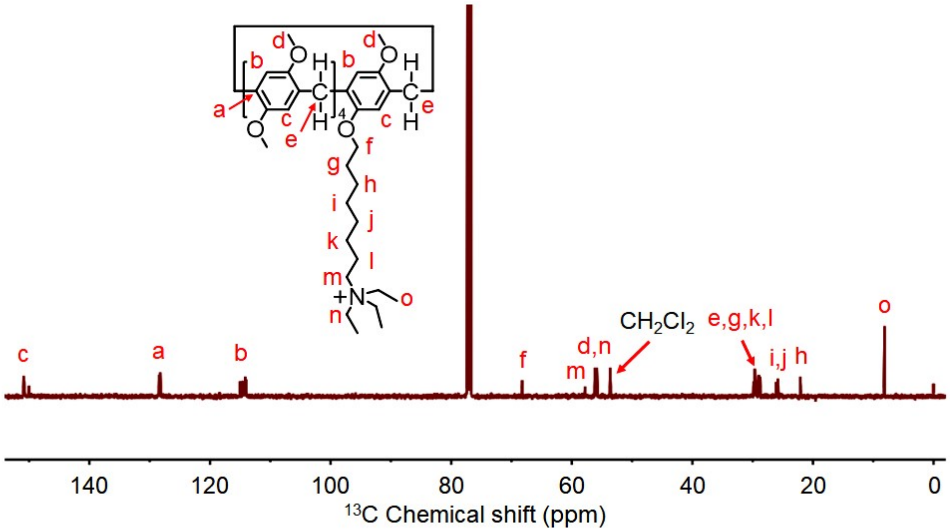

Supplementary Figure 11: ^{13}C NMR spectrum. TOP·Br in $CDCl_3$ (500 MHz, 25°C). Source data are provided as a Source Data file.

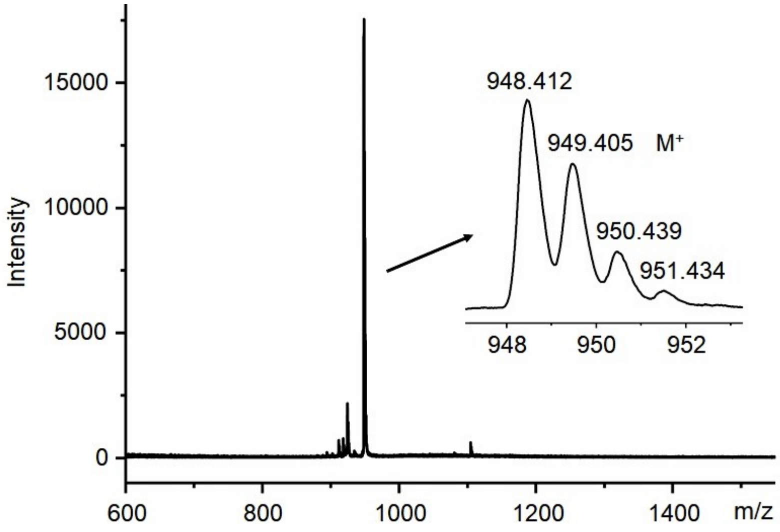

Supplementary Figure 12: MALDI-TOF MS. TOP·Br. Source data are provided as a Source Data file.

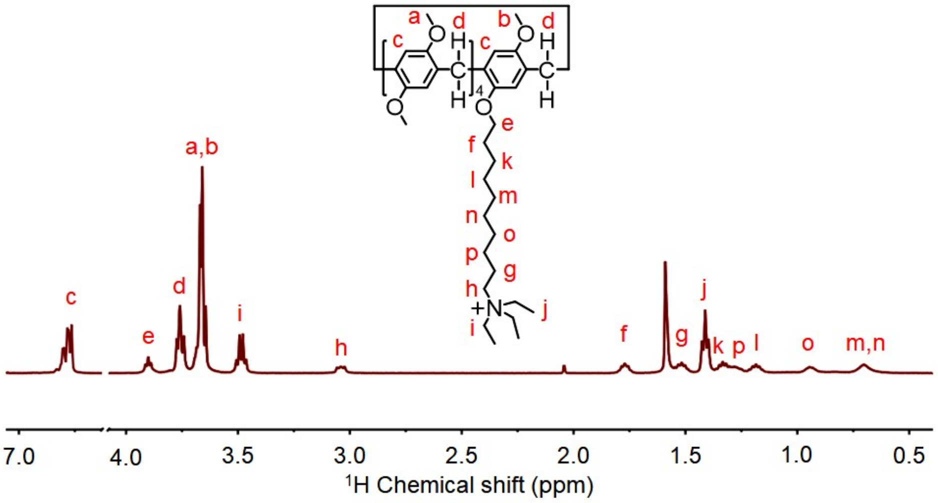

Supplementary Figure 13: ^{1}H NMR spectrum. TDP·Br in $CDCl_3$ (500 MHz, 25°C). Source data are provided as a Source Data file.

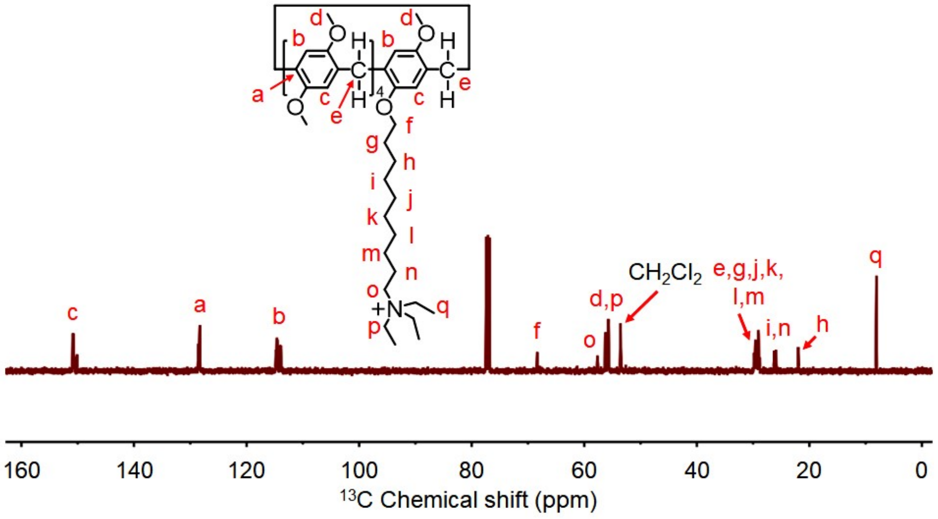

Supplementary Figure 14: ^{13}C NMR spectrum. TDP·Br in $CDCl_3$ (500 MHz, 25°C). Source data are provided as a Source Data file.

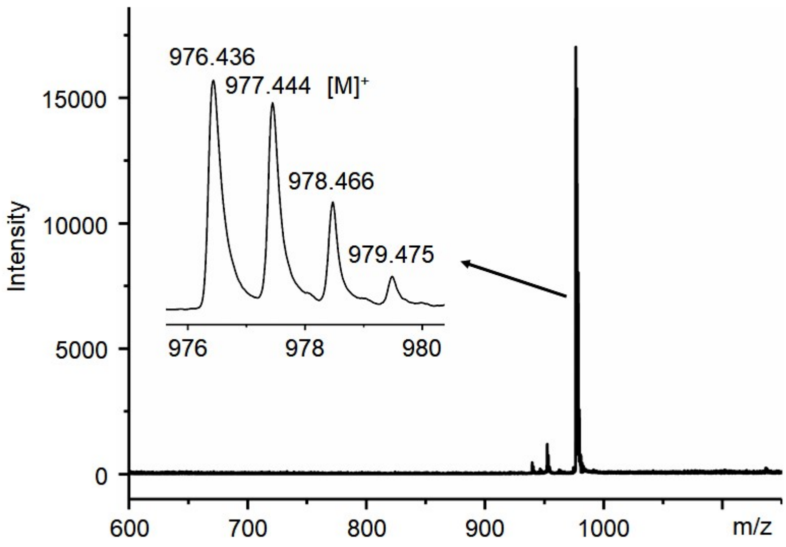

Supplementary Figure 15: MALDI-TOF MS. TDP·Br. Source data are provided as a Source Data file.

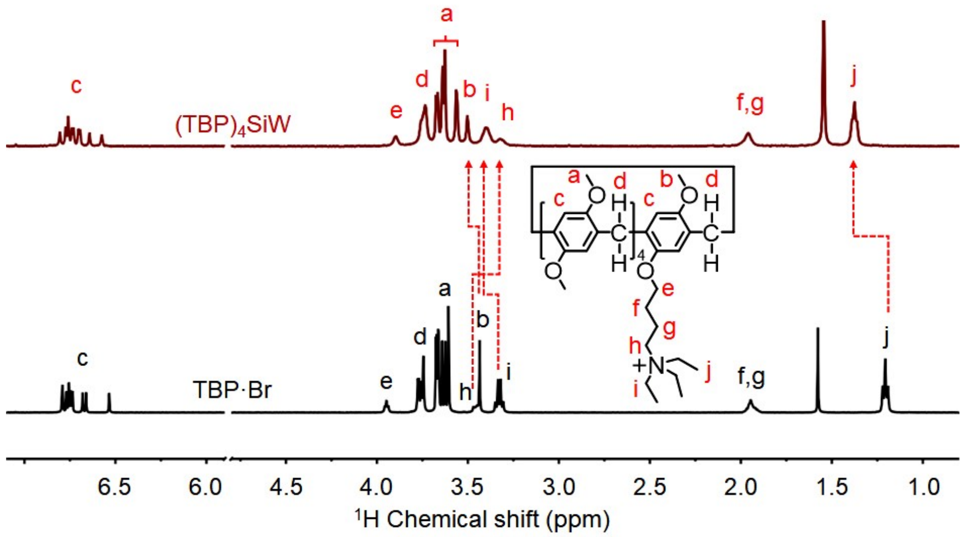

Supplementary Figure 16: ^{1}H NMR spectra. $(TBP)_4SiW$ and TBP·Br in $CDCl_3$ (500 MHz, 25°C). Source data are provided as a Source Data file.

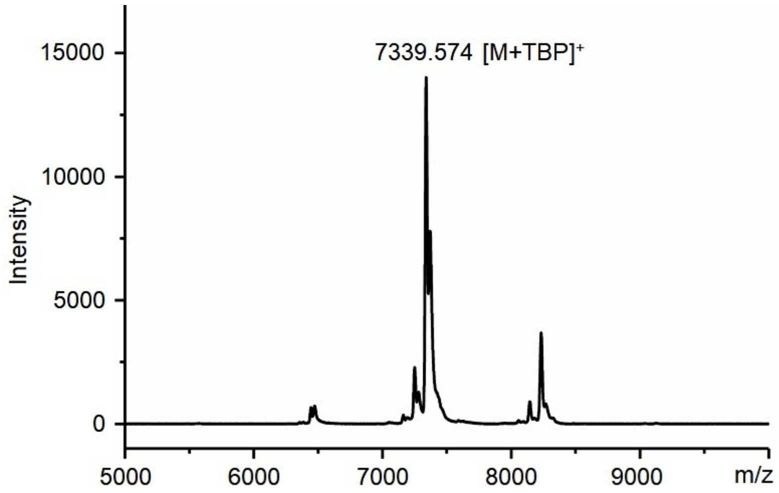

Supplementary Figure 17: MALDI-TOF MS. $(TBP)_4SiW$. Source data are provided as a Source Data file.

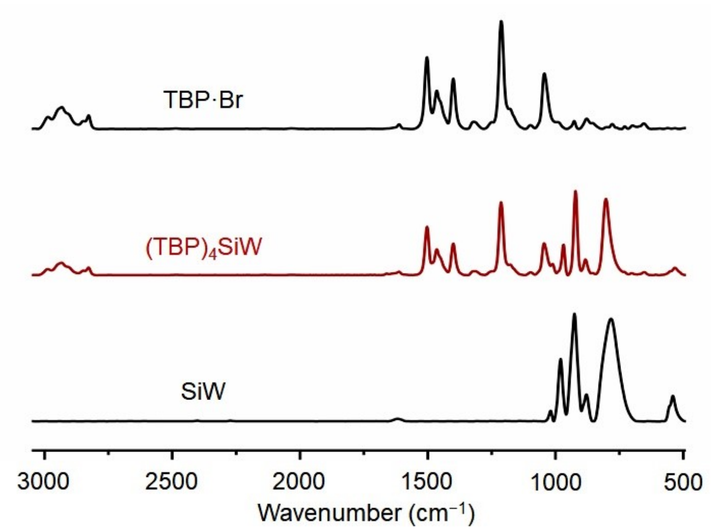

Supplementary Figure 18: FT-IR spectra. SiW, $(TBP)_4SiW$, and TBP·Br. Source data are provided as a Source Data file.

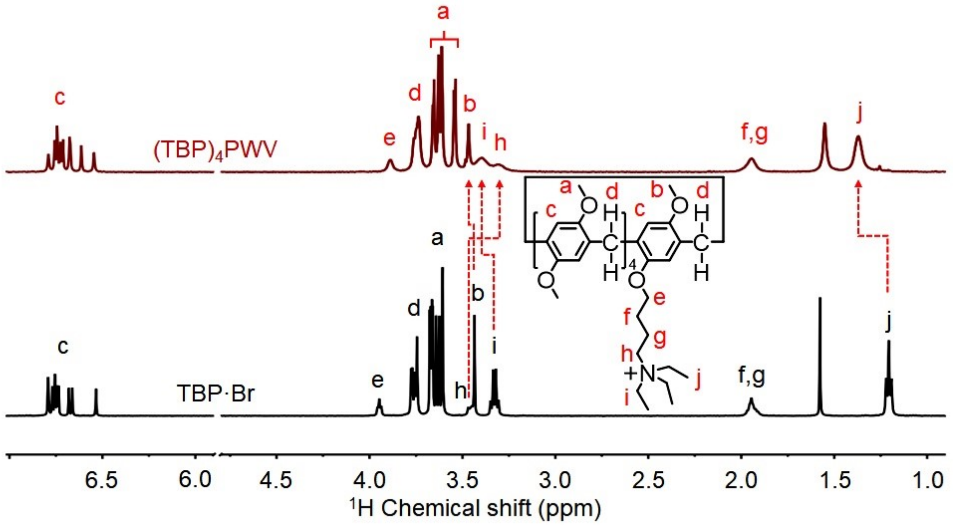

Supplementary Figure 19: ^{1}H NMR spectra. $(TBP)_4PWV$ and TBP·Br in $CDCl_3$ (500 MHz, 25°C). Source data are provided as a Source Data file.

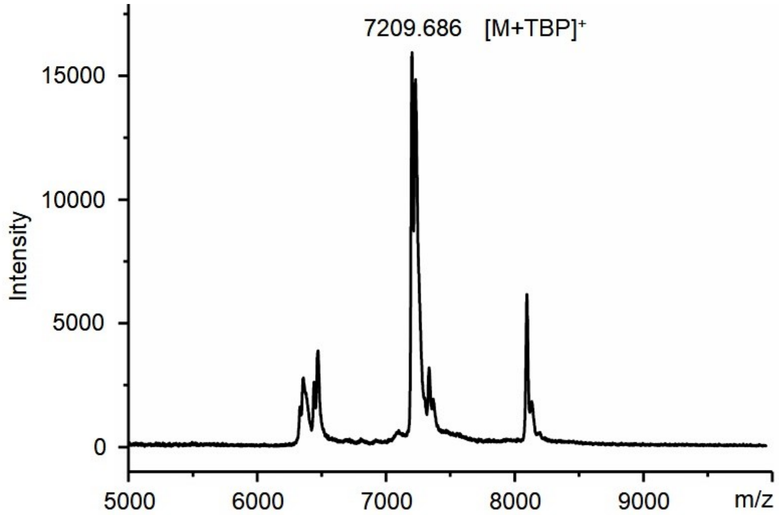

Supplementary Figure 20: MALDI-TOF MS. $(TBP)_4PWV$. Source data are provided as a Source Data file.

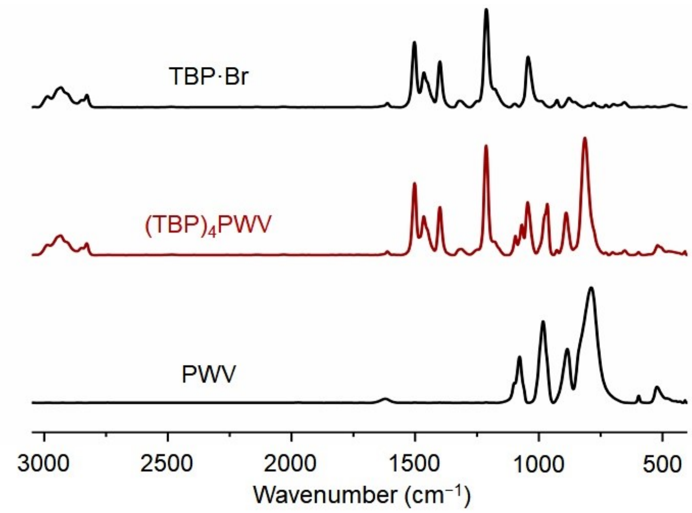

Supplementary Figure 21: FT-IR spectra. PWV, $(TBP)_4PWV$ and TBP·Br. Source data are provided as a Source Data file.

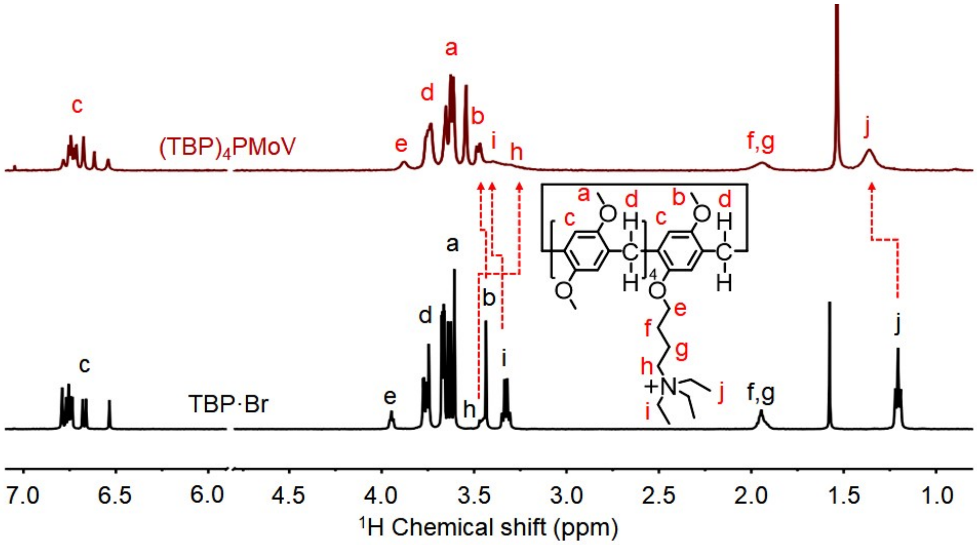

Supplementary Figure 22: ¹H NMR spectra. $(TBP)_4PMoV$ and TBP·Br in $CDCl_3$ (500 MHz, 25°C). Source data are provided as a Source Data file.

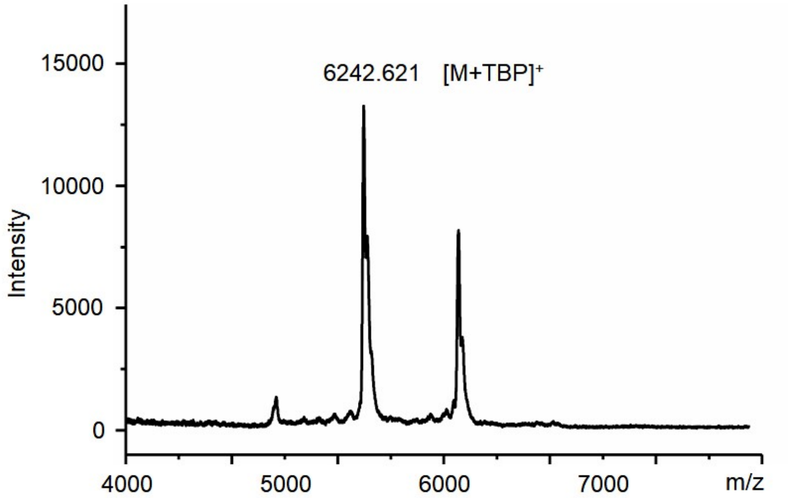

Supplementary Figure 23: MALDI-TOF MS. $(TBP)_4PMoV$. Source data are provided as a Source Data file.

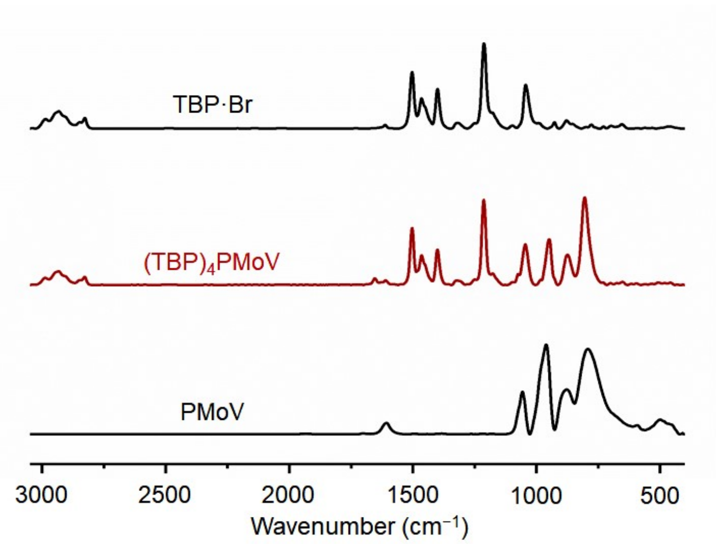

Supplementary Figure 24: FT-IR spectra. PMoV, $(TBP)_4PMoV$ and TBP·Br. Source data are provided as a Source Data file.

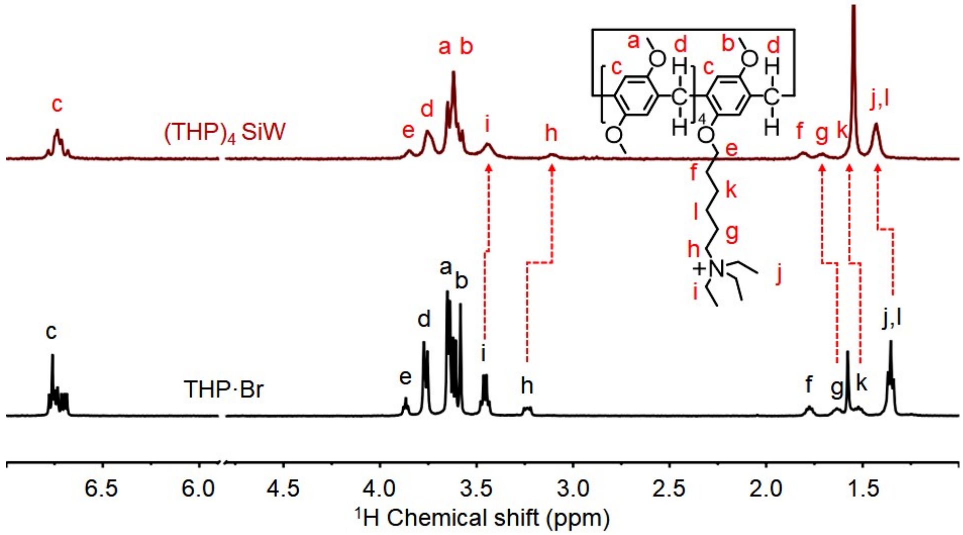

Supplementary Figure 25: ^{1}H NMR spectra. $(THP)_4SiW$ (peak of H_k and H_2O was overlap) and THP·Br in $CDCl_3$ (500 MHz, 25°C). Source data are provided as a Source Data file.

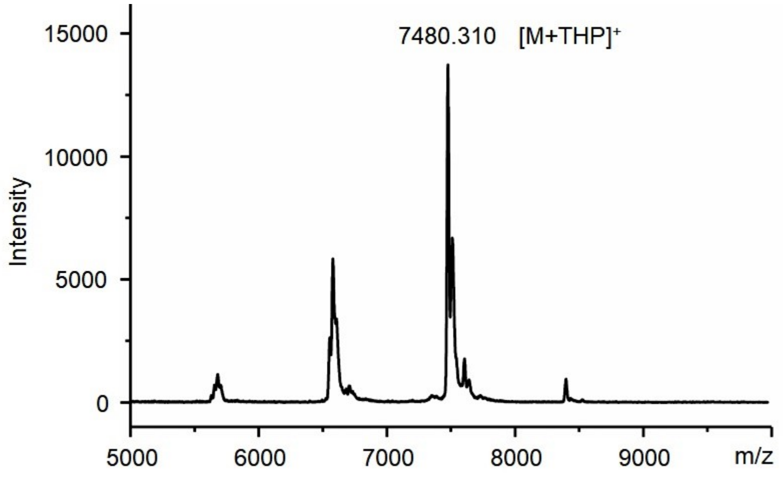

Supplementary Figure 26: MALDI-TOF MS. $(THP)_4SiW$. Source data are provided as a Source Data file.

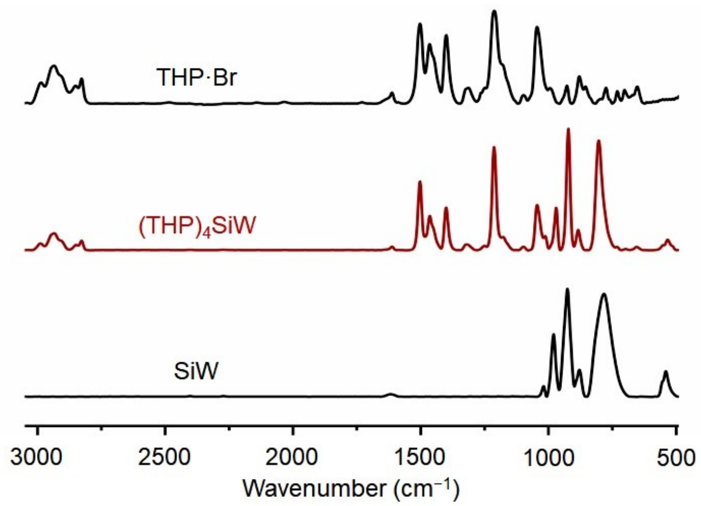

Supplementary Figure 27: FT-IR spectra. SiW, $(THP)_4SiW$ and THP·Br. Source data are provided as a Source Data file.

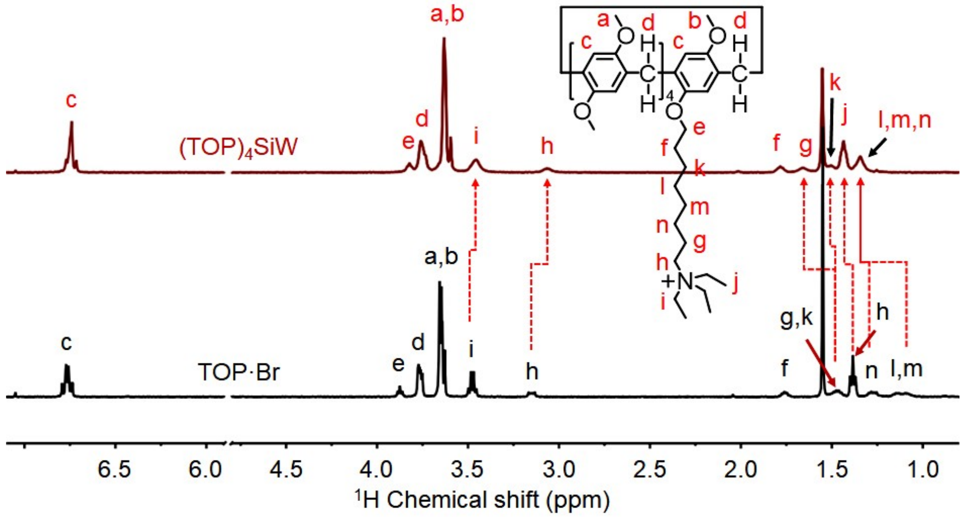

Supplementary Figure 28: ^{1}H NMR spectra. $(TOP)_4SiW$ and TOP·Br in $CDCl_3$ (500 MHz, 25°C). Source data are provided as a Source Data file.

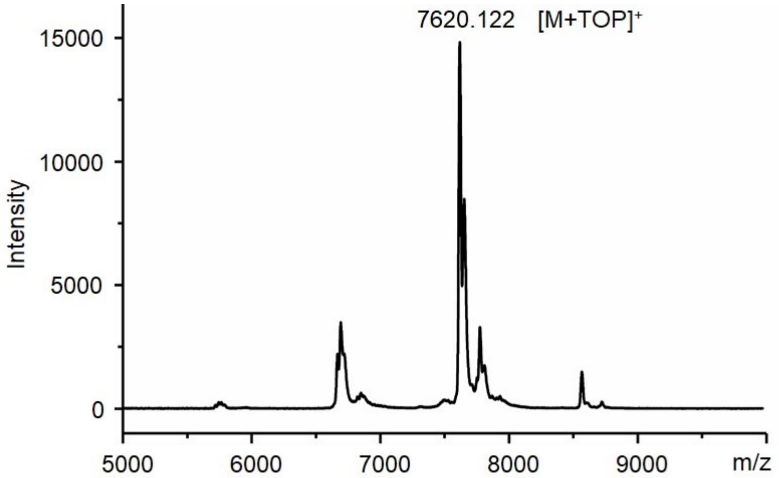

Supplementary Figure 29: MALDI-TOF MS. $(TOP)_4SiW$. Source data are provided as a Source Data file.

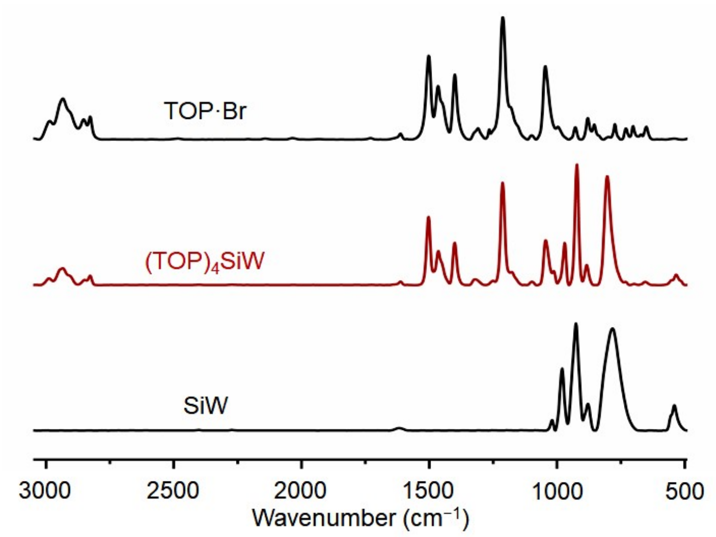

Supplementary Figure 30: FT-IR spectra. SiW, $(TOP)_4SiW$ and TOP·Br. Source data are provided as a Source Data file.

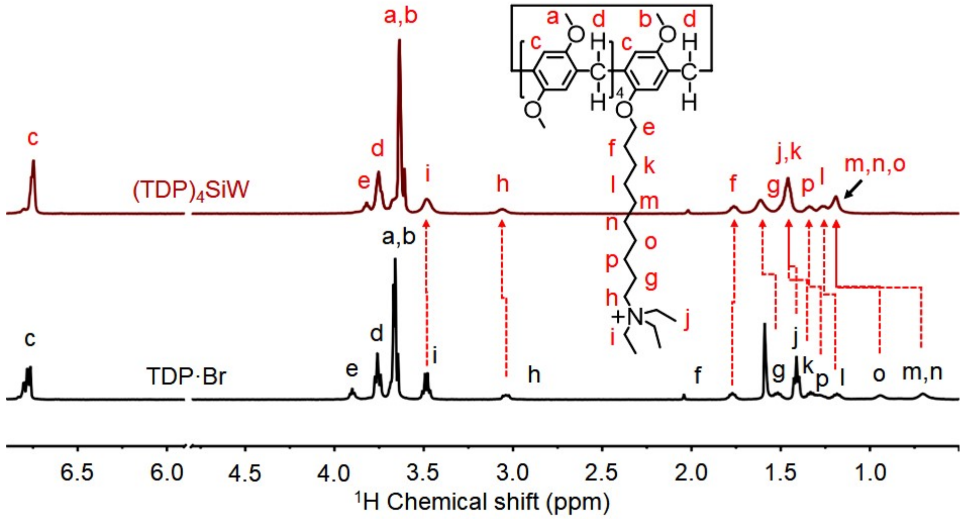

Supplementary Figure 31: ^1H NMR spectra. $(TDP)_4SiW$ (peak of H_g and H_2O was overlap) and TDP·Br in $CDCl_3$ (500 MHz, 25°C). Source data are provided as a Source Data file.

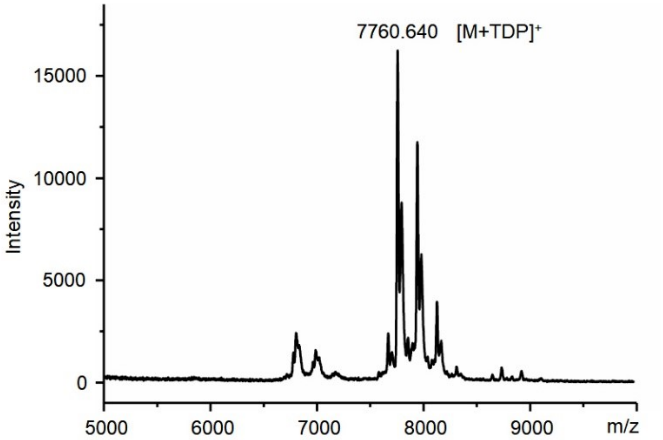

Supplementary Figure 32: MALDI-TOF MS. $(TDP)_4SiW$. Source data are provided as a Source Data file.

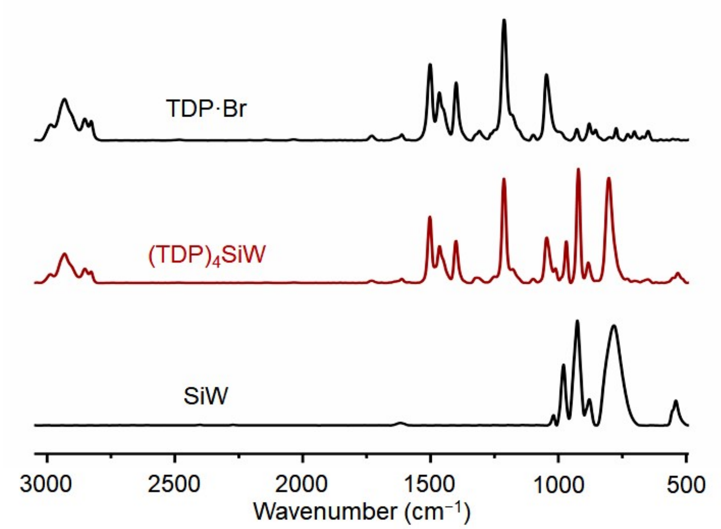

Supplementary Figure 33: FT-IR spectra. SiW, $(TDP)_4SiW$ and TDP·Br. Source data are provided as a Source Data file.

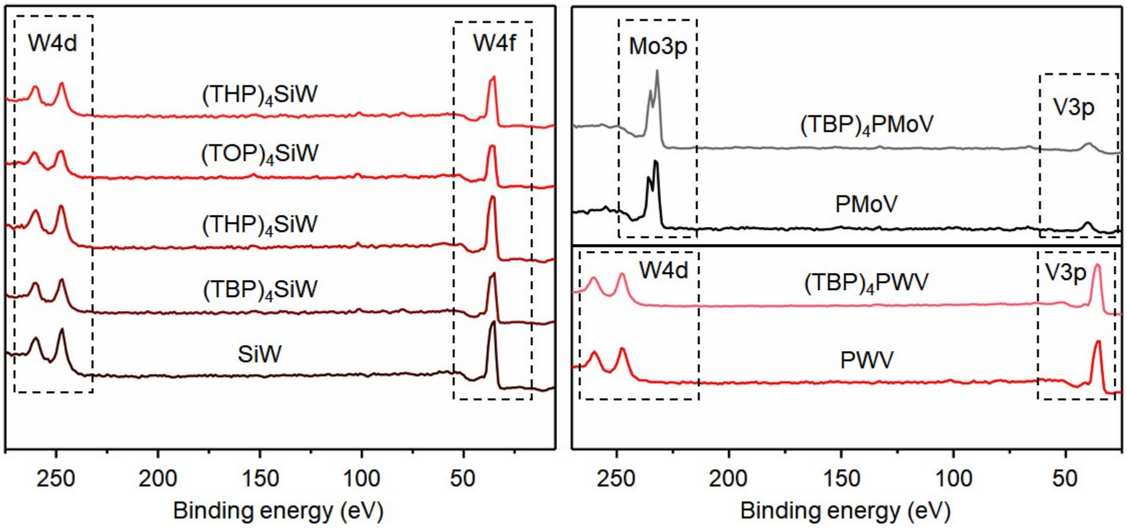

Supplementary Figure 34: XPS. SiW, $(TBP)_4SiW$, $(THP)_4SiW$, $(TOP)_4SiW$, $(TDP)_4SiW$, PWV, $(TBP)_4PWV$, PMoV, and $(TBP)_4PMoV$. The W(4d), Mo(3p), V(3p) peeks has no obvious shift demonstrate the stability of POM before and after ion-exchange. Source data are provided as a Source Data file.

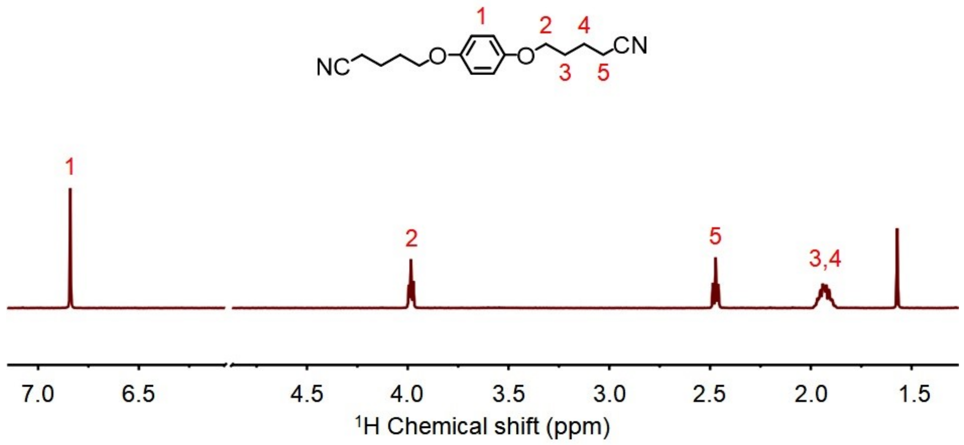

Supplementary Figure 35: ^{1}H NMR spectrum. BCB in $CDCl_3$ (500 MHz, 25°C). Source data are provided as a Source Data file.

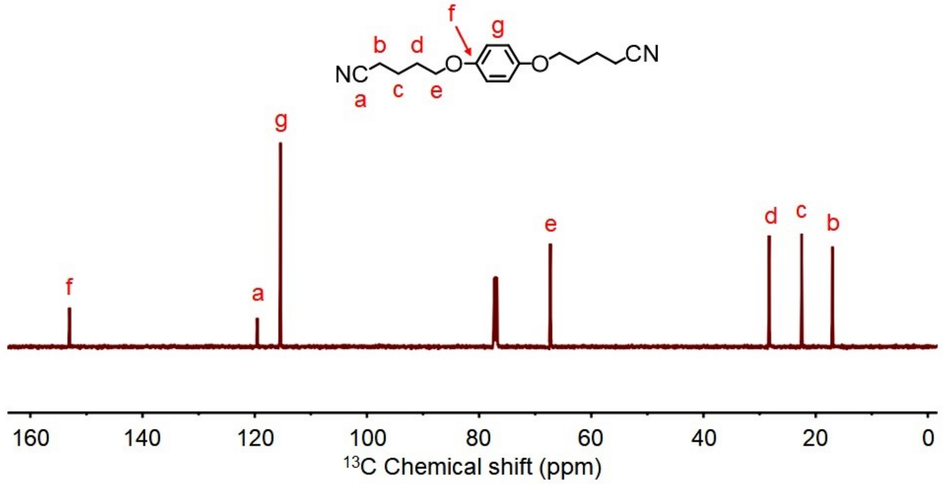

Supplementary Figure 36: ^{13}C NMR spectrum. BCB in $CDCl_3$ (500 MHz, 25°C). Source data are provided as a Source Data file.

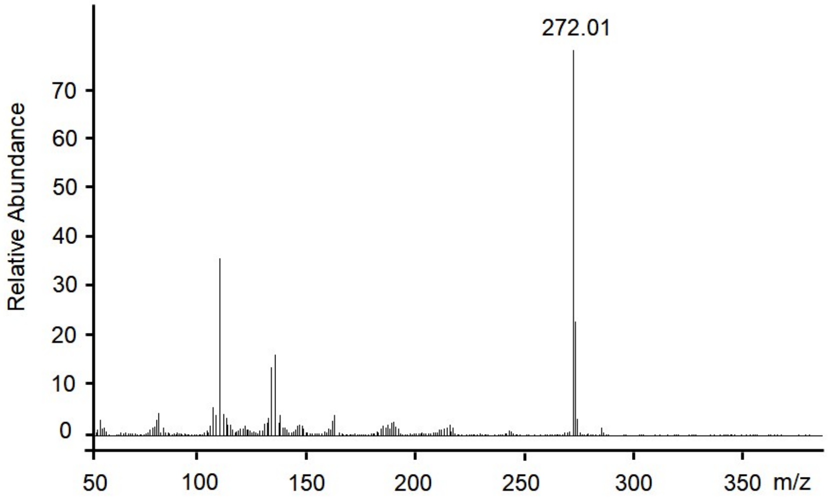

Supplementary Figure 37: GC MS. BCB. Source data are provided as a Source Data file.

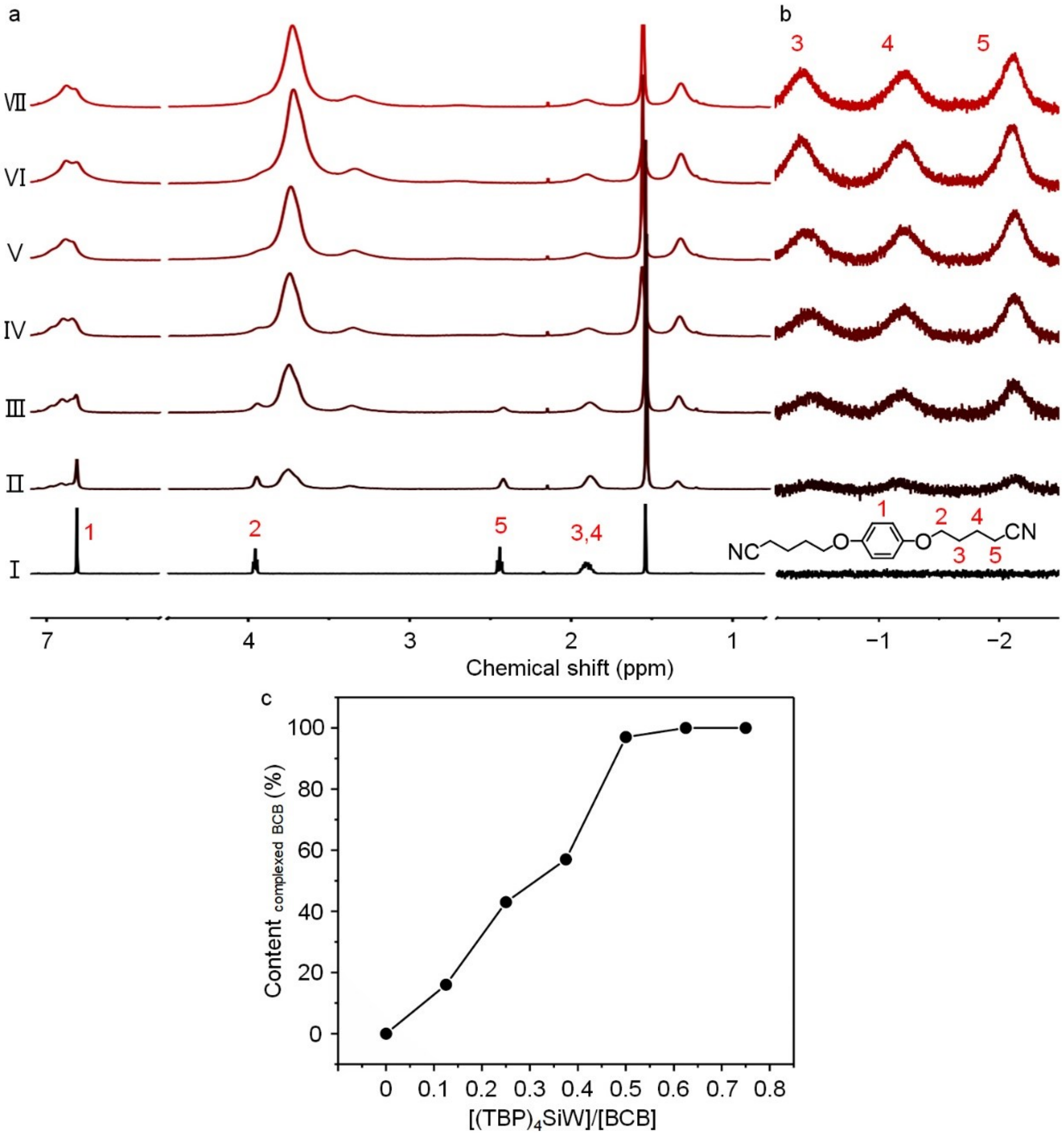

Supplementary Figure 38: Partial ^{1}H NMR spectra ($CDCl_3$, 500 MHz, 25°C) of (I) BCB (4 mM) upon addition (II) 0, (III) 0.125, (IV) 0.25, (V) 0.375, (VI) 0.5, (VII) 0.625, (VIII) 0.75 eq. $(TBP)_4SiW$. **(a)** Chemical shift: 7.1−0.8 ppm; **(b)** Increased signals of chemical shift: (−0.08) − (−2.5) ppm. **(c)** Plot of relative content of complexed DDB calculated from integral area of H_5 versus the molar ratio of $(TBP)_4SiW$ to BCB, used TMS as internal standard. Source data are provided as a Source Data file.

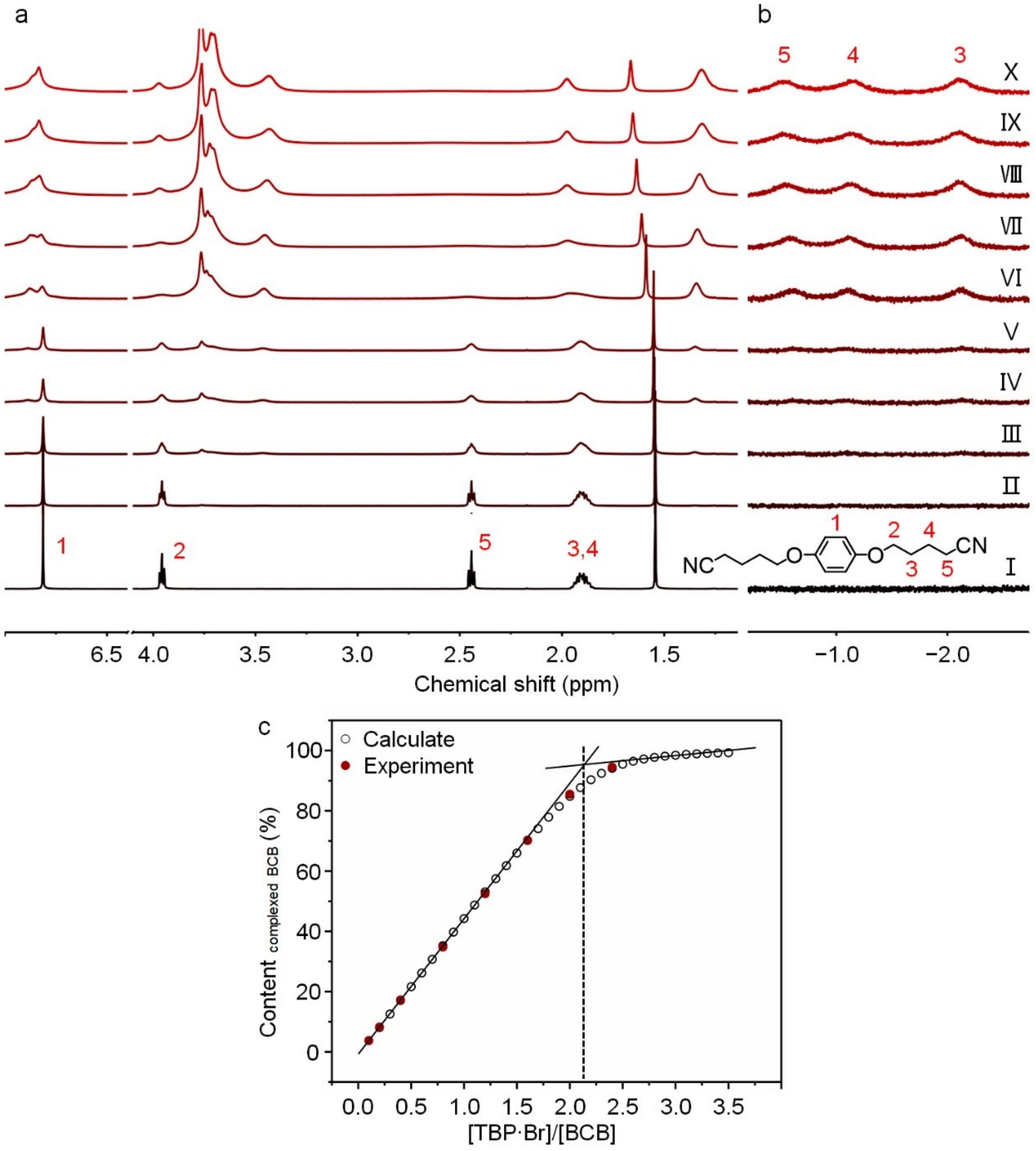

Supplementary Figure 39: Partial ^1H NMR spectra ($CDCl_3$, 500 MHz, 25°C) of BCB (10 mM) upon addition (I) 0, (II 0.1, (III) 0.2, (IV) 0.4, (V) 0.6, (VI) 0.8, (VII) 1.2, (VIII) 1.6, (IX) 2.0, (X) 2.4 eq. TBP·Br: **(a)** Chemical shift: 7.0−1.1 ppm, **(b)** Increased signals of chemical shift: 0− (−2.5) ppm. **(c)** Plot of relative content of complex G calculated from integral area of H_5 versus the molar ratio of TBP·Br to BCB, used TMS as internal standard. Source data are provided as a Source Data file.

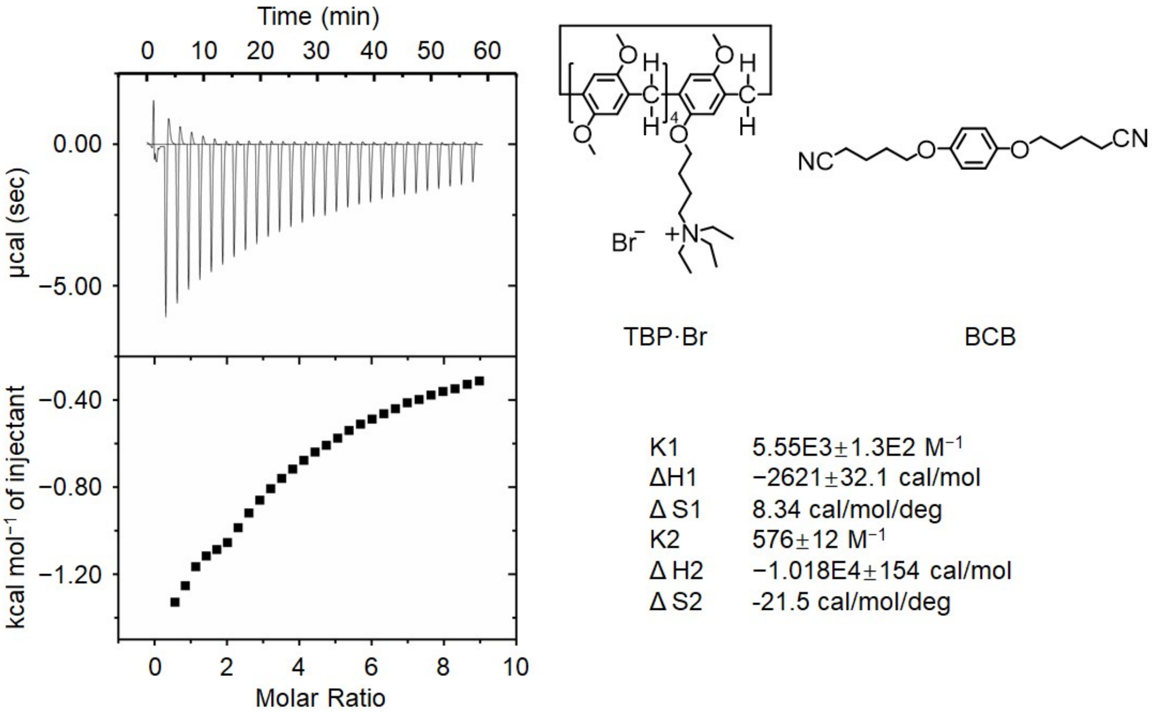

Supplementary Figure 40: ITC titration curve and fitted data of TBP·Br to BCB in chloroform at 25°C, which gave the 2:1 association constant K_1=5.55±0.13×10^3 M^{-1} and K_2= 5.76±0.12×10^2 M^{-1}. "±" values represent standard deviations. Source data are provided as a Source Data file.

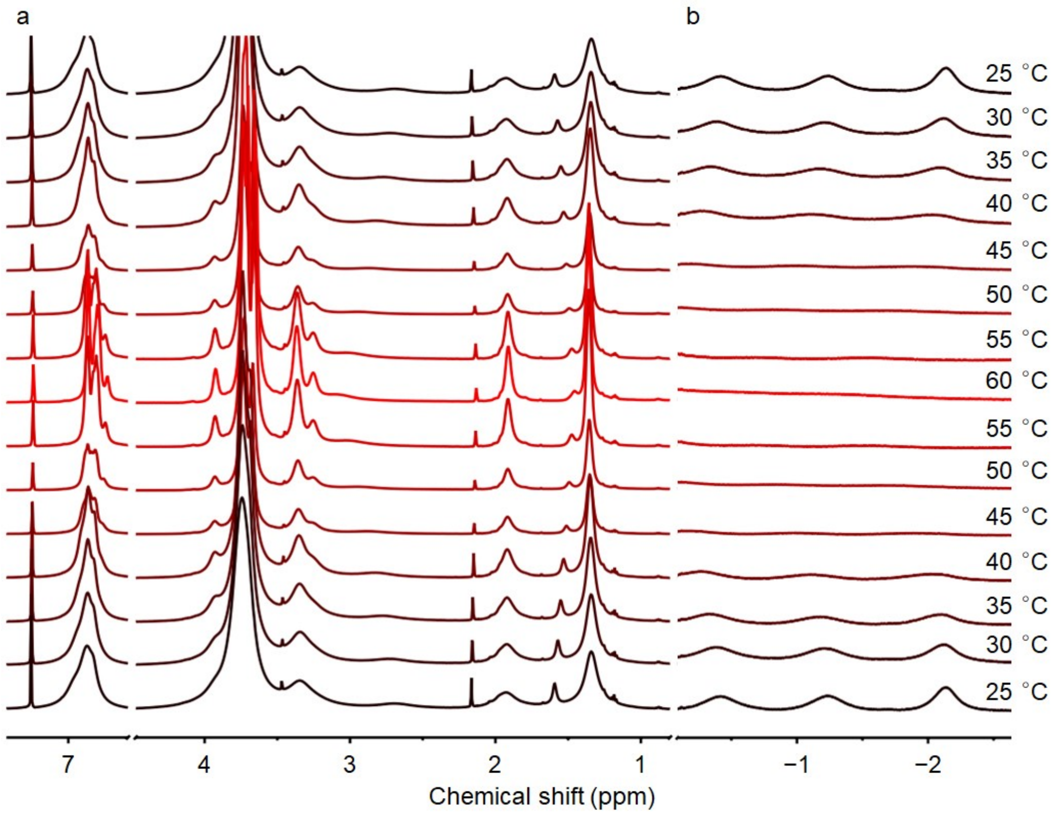

Supplementary Figure 41: Partial ^{1}H NMR spectra ($CDCl_3$, 500 MHz) of $(TBP)_4$SiW@BCB [1:2, 4 mM, based on $(TBP)_4$SiW] at variable temperature. **(a)** Chemical shift: 7.1−0.9 ppm; **(b)** Increased signals of chemical shift: (−0.08) − (−2.5) ppm. Source data are provided as a Source Data file.

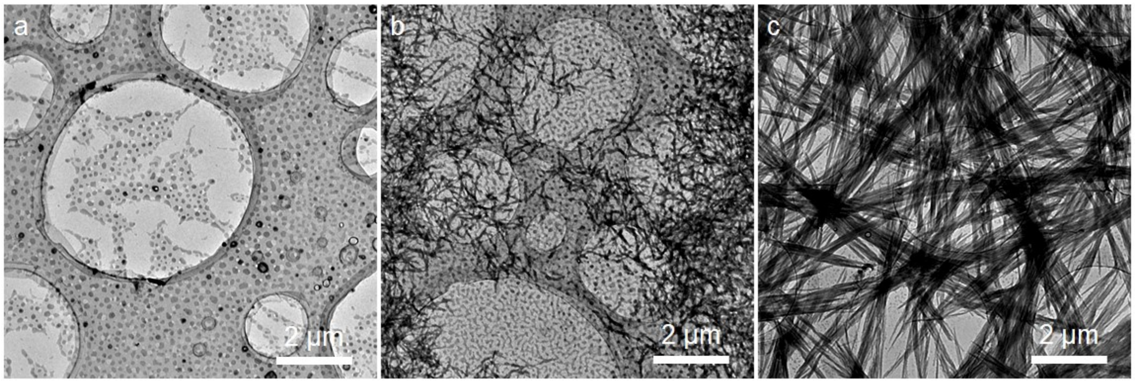

Supplementary Figure 42: TEM images of $(TBP)_4SiW@BCB$ (1:2) in $CHCl_3$ at concentration of **(a)** 0.08 mM, **(b)** 0.15 mM, and **(c)** 0.25 mM, after 3 h of sonication at 20°C. Source data are provided as a Source Data file.

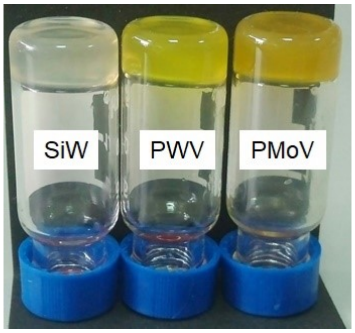

Supplementary Figure 43: Digital pictures of gels constructed from different polyoxometalates (4 mM based on ionic complex hosts). Source data are provided as a Source Data file.

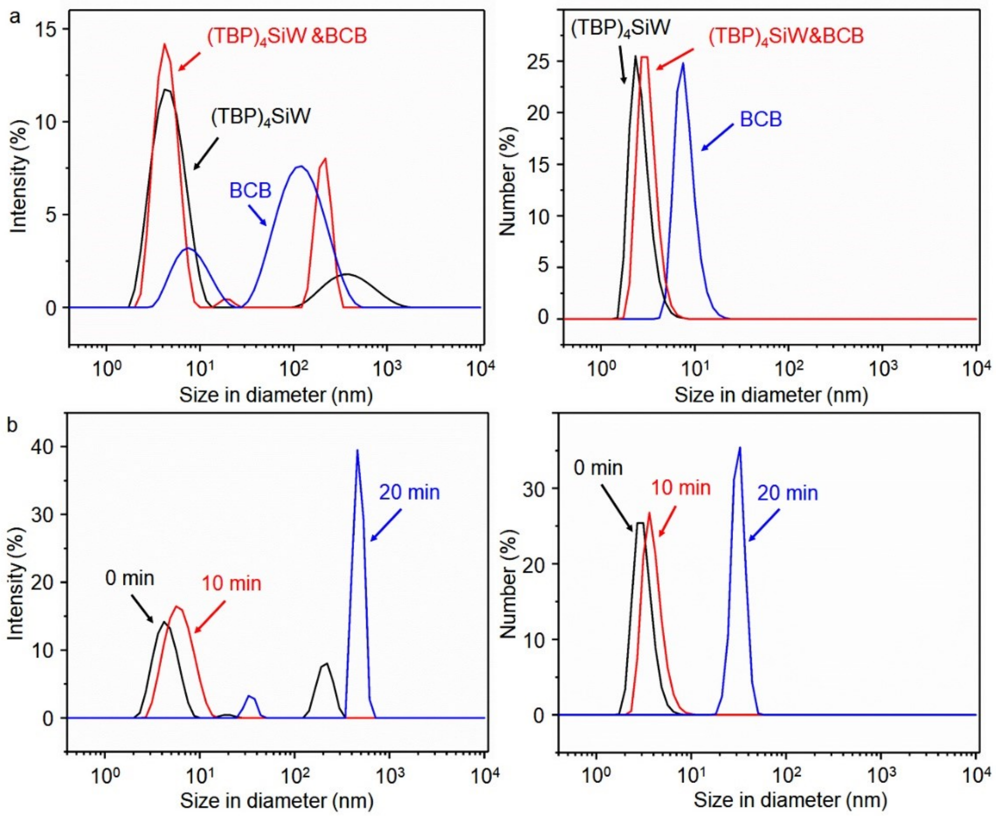

Supplementary Figure 44: DLS date of **(a)**, $(TBP)_4SiW$ (0.25 mM), $(TBP)_4SiW@BCB$ (1:2, 0.25 mM), BCB (0.5 mM) in $CHCl_3$ at 20 °C, and **(b)**, $(TBP)_4SiW@BCB$ (1:2, 0.25 mM) with different sonication time at 20 °C. Source data are provided as a Source Data file.

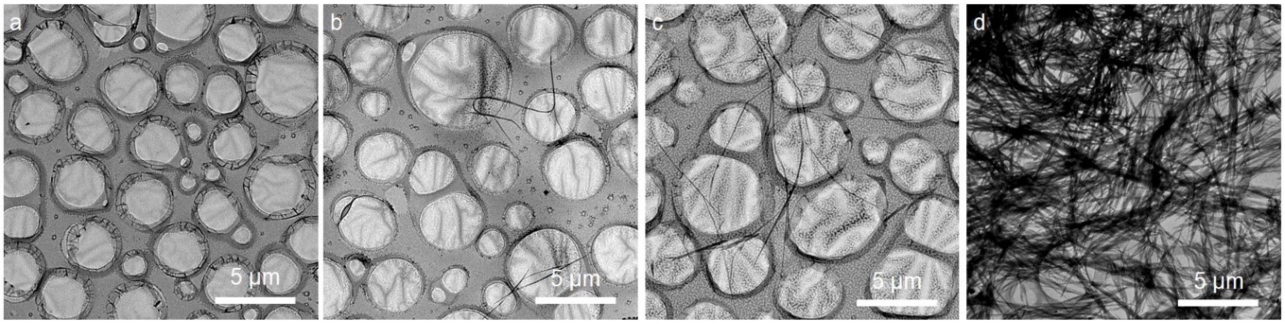

Supplementary Figure 45: TEM images of $(TBP)_4SiW@BCB$ (1:2, 0.25 mM) in $CHCl_3$ with different sonication treatment, **(a)** 0 h; **(b)** 1 h; **(c)** 2 h; **(d)** 3 h. Source data are provided as a Source Data file.

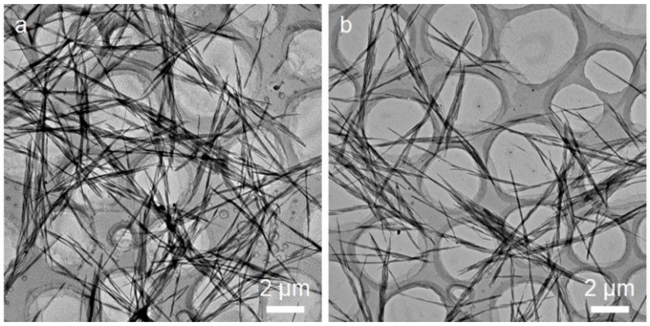

Supplementary Figure 46: TEM images of the dilute $(TBP)_4SiW@BCB$ gel solution with different concentrations: (a) 0.05 mM and (b) 0.01 mM based on $[(TBP)_4SiW]$. Source data are provided as a Source Data file.

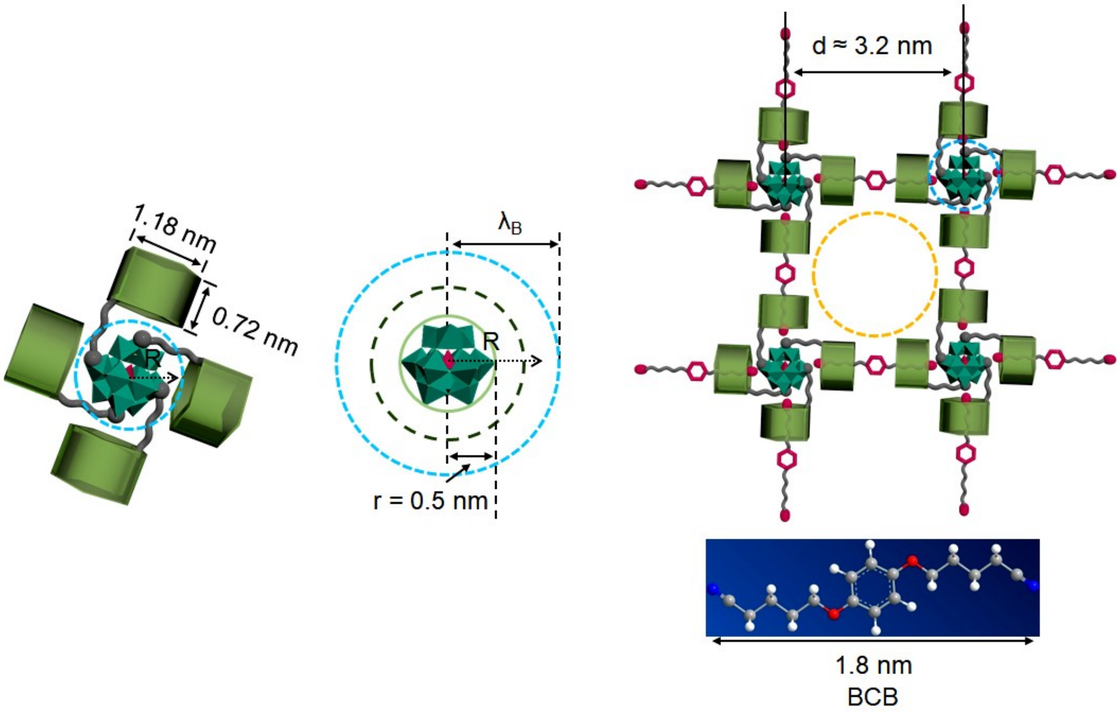

Supplementary Figure 47: Structural matching relation of four surfactants around one POM. In solvents, ion pairs are limited in the Bjerrum length λ_{B}.[4] $\lambda_{\mathrm{B}} = e^2/4\pi\varepsilon_0\varepsilon_S k_B T$, e is the elementary charge, ε_0 the vacuum permittivity, ε_S the dielectric permittivity of the surrounding solvent, k_B is the Boltzmann constant, and T is the absolute temperature. In water, the Bjerrum length is 0.7 nm, however, it is 11.7 nm in chloroform. $S_{P5} = \pi r^2 = 3.14 \times (1.18/2)^2 = 1.09\ \mathrm{nm}^2$, $4S_{P5} = 4.36\ \mathrm{nm}^2$, If four surfactants can around one POM, then $4\pi\mathrm{R}^2 \geq 4S_{P5}$, so $\mathrm{R} \geq 0.59\ \mathrm{nm}$, R is the distance of POM and pillar[5]arene. While $0.7\ \mathrm{nm} \leq \mathrm{R} \leq \lambda_B \approx 11.7\ \mathrm{nm}$, which is include the region of above mentioned. $d = 2\mathrm{R} + l_{BCB} = 3.2\ \mathrm{nm}$, *d* is the distance of two POMs, and the pore size is about 1.8 nm.

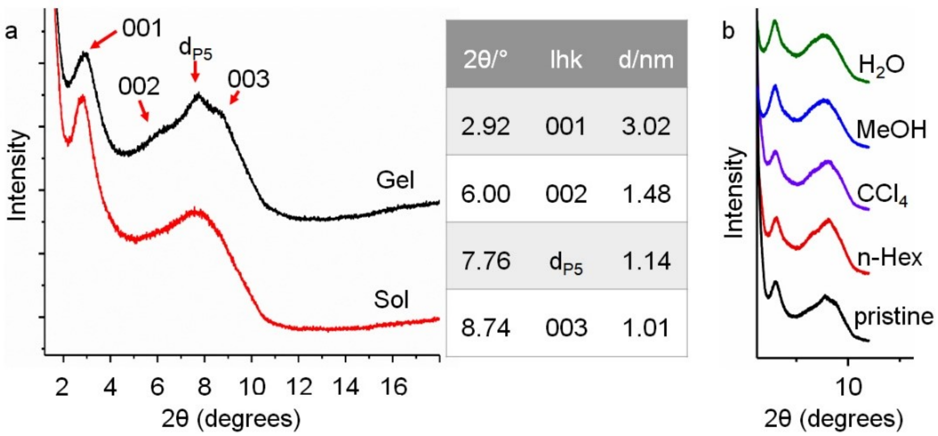

2θ/°	lhk	d/nm
2.92	001	3.02
6.00	002	1.48
7.76	d_{P5}	1.14
8.74	003	1.01

Supplementary Figure 48: (a) Powder XRD of prepared supramolecular gel and its initial sol state of $(TBP)_4SiW@BCB$ (d_{P5} is the diameter of methyl-pillar[5]arene). **(b)** Powder XRD of $(TBP)_4SiW@BCB$ after treatment with different solvents for one week. Source data are provided as a Source Data file.

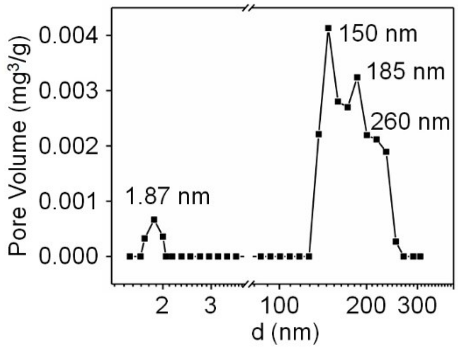

Supplementary Figure 49: Pore size distribution calculated by both NLDFT method and BET method from N_2 sorption isotherms for $(TBP)_4SiW@BCB$ gel powder at 77K. It shows the micropore size at approximately 1.87 nm, and the macropore size over 150 nm. Source data are provided as a Source Data file.

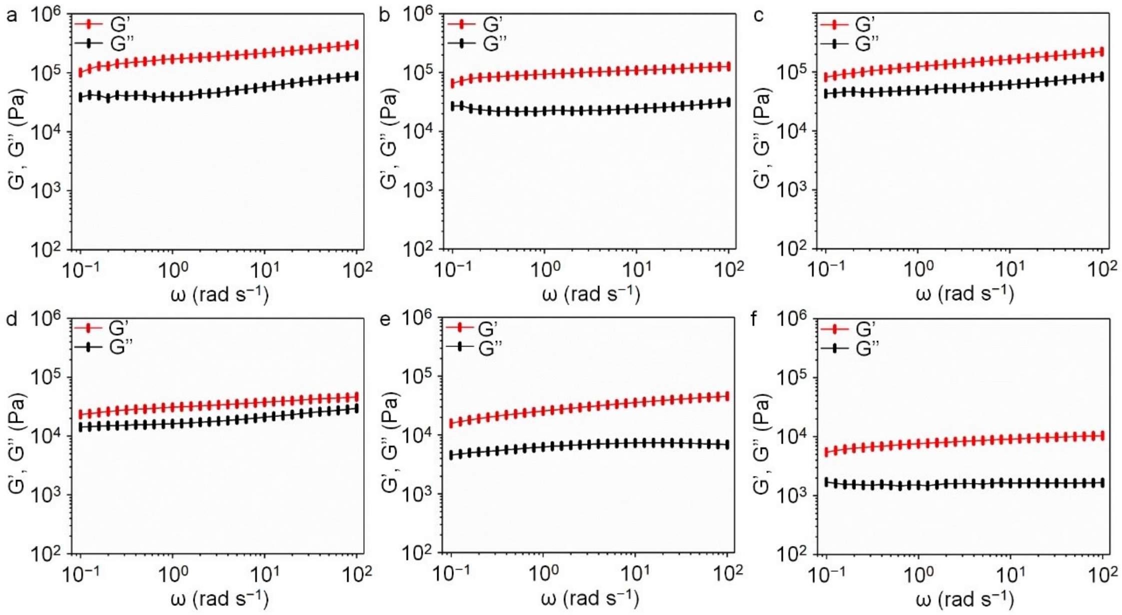

Supplementary Figure 50: Angular frequency (ω) dependence of storage and loss moduli, G' and G'', with a cyclic oscillation amplitude of 0.5%, at 25°C for gels: **(a)** $(TBP)_4SiW@BCB$, **(b)** $(TBP)_4PWV@BCB$, **(c)** $(TBP)_4PMoV@BCB$, **(d)** $(THP)_4SiW@BCB$, **(e)** $(TOP)_4SiW@BCB$, **(f)** $(TDP)_4SiW@BCB$. Source data are provided as a Source Data file.

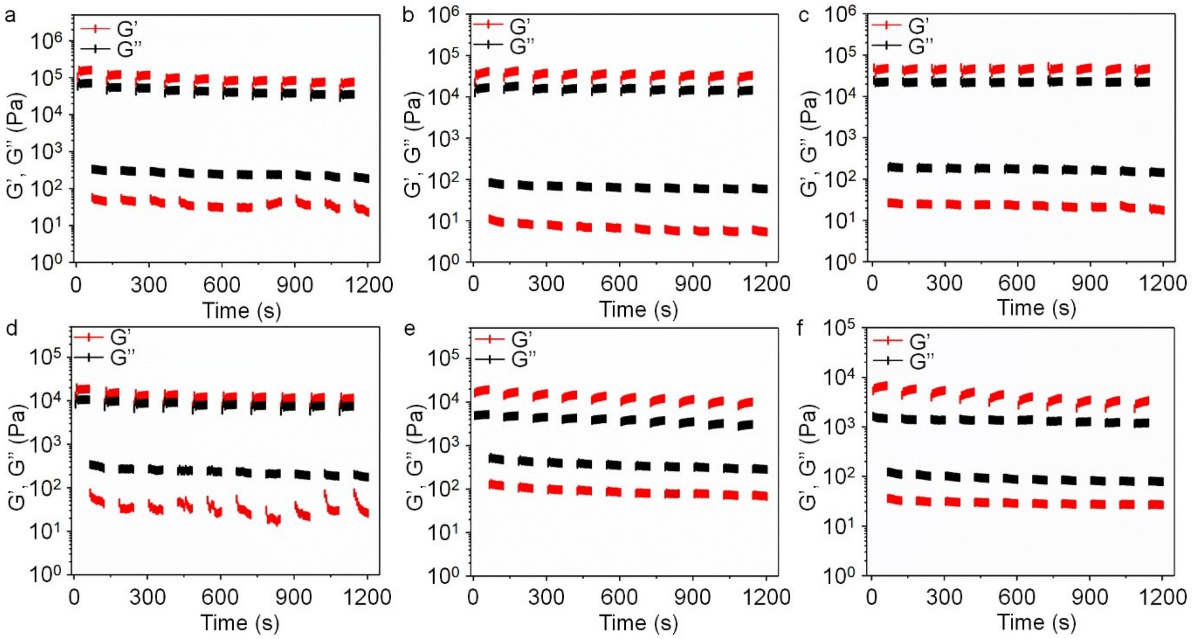

Supplementary Figure 51: Angular frequency (ω) dependence of storage and loss moduli, G' and G'', of the gel under a cyclic oscillation amplitude between 0.5% and 100% at 25°C with ω= 10 rad s^{-1}. **(a)** $(TBP)_4SiW@BCB$, **(b)** $(TBP)_4PWV@BCB$, **(c)** $(TBP)_4PMoV@BCB$, **(d)** $(THP)_4SiW@BCB$, **(e)** $(TOP)_4SiW@BCB$, **(f)** $(TDP)_4SiW@BCB$. Source data are provided as a Source Data file.

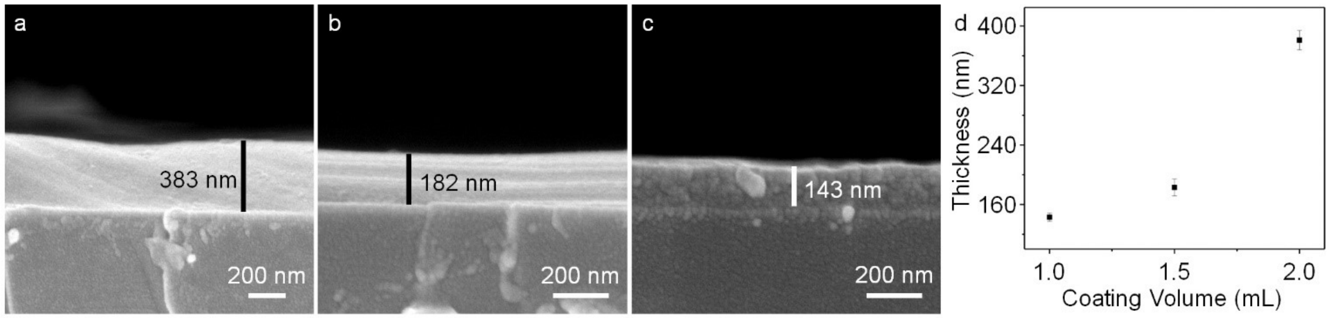

Supplementary Figure 52: Cross-sectional SEM images of coating membranes with different coating volume: **(a)** 2 mL, **(b)** 1.5 mL, **(c)** 1.0 mL of dilute $(TBP)_4SiW@BCB$ gel solution [1 mM, based on $(TBP)_4SiW$]. **(d)** Work plot of thickness versus the coating volume. Source data are provided as a Source Data file.

Supplementary Table 1: Summary of the influence from solvent on membrane separation.

liquids	Membrane behavior	Membrane stability	Joystick liquid
Pure water	unchanged	Yes	No
Saturated NaCl solution	unchanged	Yes	No
Aqueous solution (HCl, pH=1)	unchanged	Yes	No
Aqueous solution (NaOH, pH=12)	unchanged	Yes	No
Ethylene glycol	unchanged	Yes	No
Glycerine	unchanged	Yes	No
Methanol	unchanged	Yes	Yes

Ethanol	unchanged	Yes	Yes
Isopropanol	unchanged	Yes	Yes
Tetrahydrofuran	unchanged	Yes	Yes
Acetone	shrunken	No	No
Acetonitrile	shrunken	No	No
Dimethyl sulfoxide, DMSO	dissolved	No	No
Dimethyl formamide, DMF	dissolved	No	No
Formamide	dissolved	No	No
Adiponitrile	dissolved	No	No
Dibromoethane	dissolved	No	No
Dibromopropane	dissolved	No	No
Dibromobutane	dissolved	No	No
Dichloromethane	dissolved	No	No

Supplementary Table 2: Summary of immiscible liquids that have been separated with the $(TBP)_4SiW$@BCB membrane.[a]

	H_2O	EG	GW	Diesel	Soybean Oil	Gasoline	o-DCB	EBA	CCl_4	BZ	Tol	n-Hex
**n-Hex**	√	√	√	–	–	–	–	–	–	–	–	–
**Tol**	√	√	√	–	–	–	–	–	–	–	–	–
**BZ**	√	√	√	–	–	–	–	–	–	–	–	–
**CCl_4**	√	√	√	–	–	–	–	–	–	–	–	–
**EBA**	√			–	–	–	–	–	–	–	–	–
**o-DCB**	√			–	–	–	–	–	–	–	–	–
**Diesel**	√	√	√	–	–	–	–	–	–	–	–	–
**Gasoline**	√	√	√	–	–	–	–	–	–	–	–	–
**Soybean Oil**	√	√	√	–	–	–	–	–	–	–	–	–
**GW**	–	–	–	√	√	√	√	√	√	√	√	√
**EG**	–	–	–	√	√	√	√	√	√	√	√	√
**H_2O**	–	–	–	√	√	√	√	√	√	√	√	√

[a] Symbol '√' in the squares indicates the corresponding two liquids separable while '–' indicates the corresponding two liquids miscible and inseparable, where EG: ethylene glycol; GW: glycerinated water (67.7% glycerin); o-DCB: o-dichlorobenzene; EBA: ethyl benzoate; BZ: benzene; Tol: toluene; n-Hex: n-hexane.

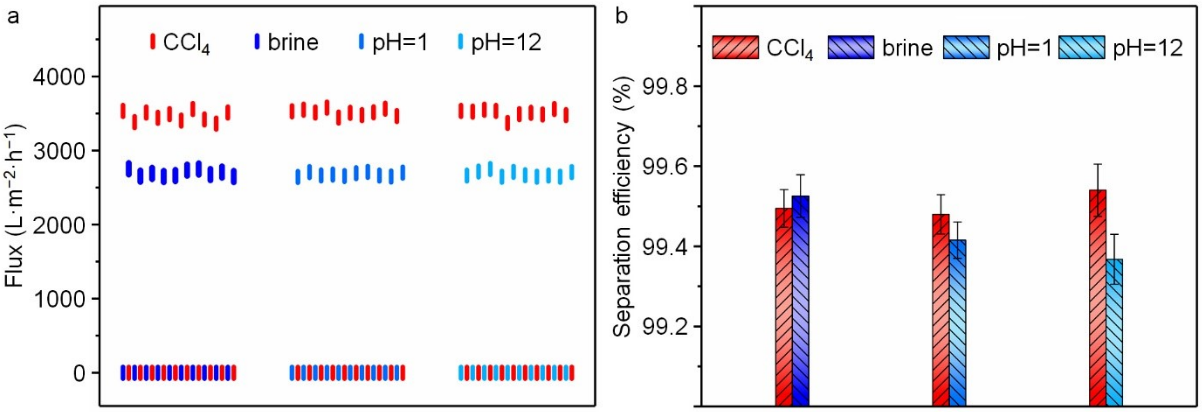

Supplementary Figure 53: (a) Flux and **(b)** separation efficiency data of the membranes for the separation of CCl_4 and aqueous solution under gravity (10 switching cycles), where brine is saturated sodium chloride aqueous solution, pH=1 is hydrochloric acid solution, and pH=12 is sodium hydroxide solution. Source data are provided as a Source Data file.

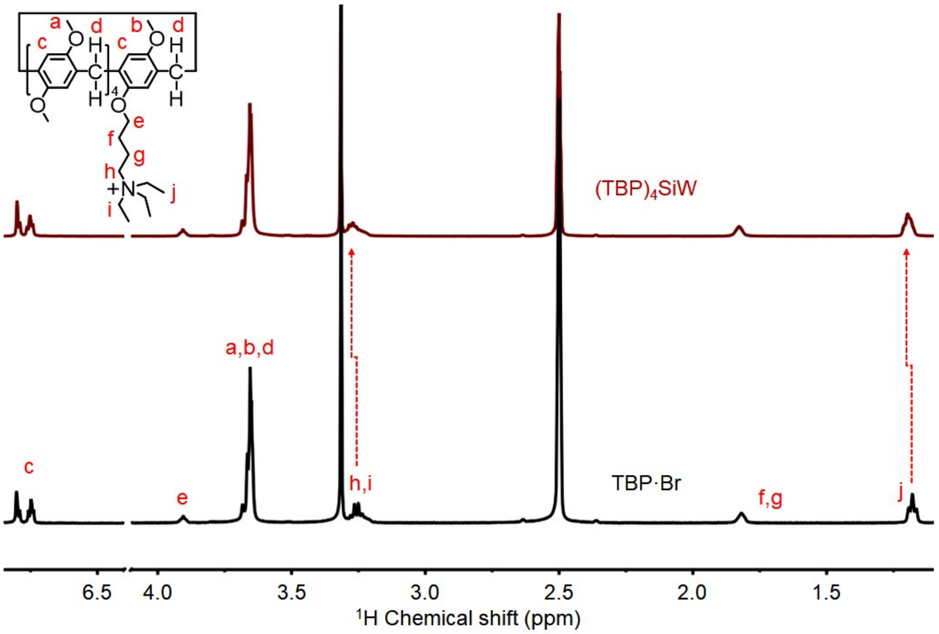

Supplementary Figure 54: Partial ^1H NMR spectra (500 MHz, 25°C) of $(TBP)_4SiW$ and TBP·Br in DMSO. Source data are provided as a Source Data file.

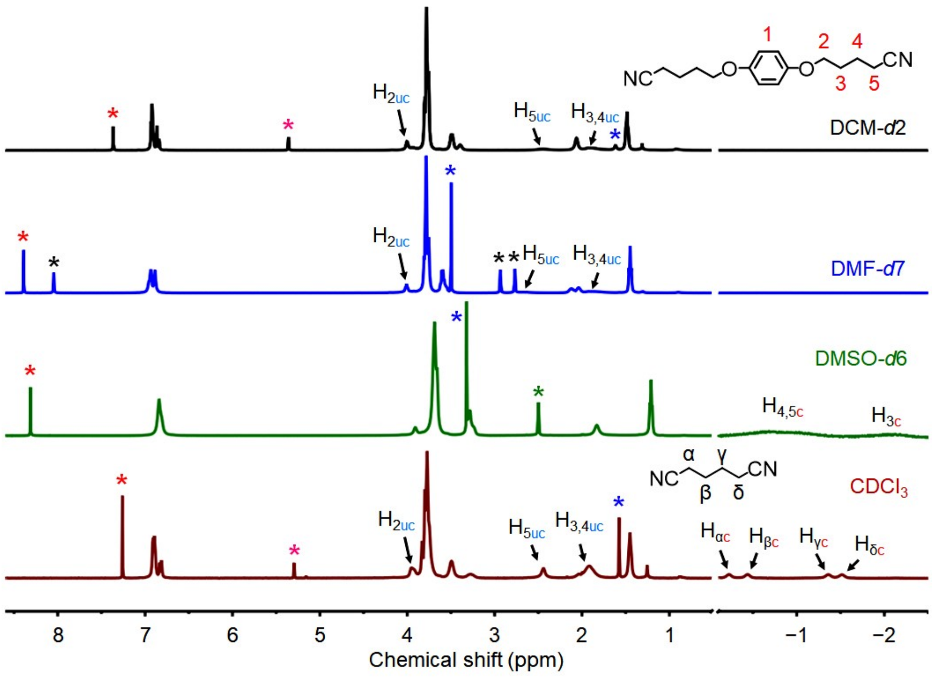

Supplementary Figure 55: Partial ^1H NMR spectra (500 MHz, 25°C) of $(TBP)_4SiW$@BCB gel in different solvents or with competitive guest (ADN), where * is the peak of water; * is the peak of chloroform; * is the peak of dichloromethane; * is the peak of dimethyl formamide (DMF); * is the peak of dimethylsulfoxide (DMSO); **c**, represents complex state; **uc**, represents uncomplex state. Source data are provided as a Source Data file.

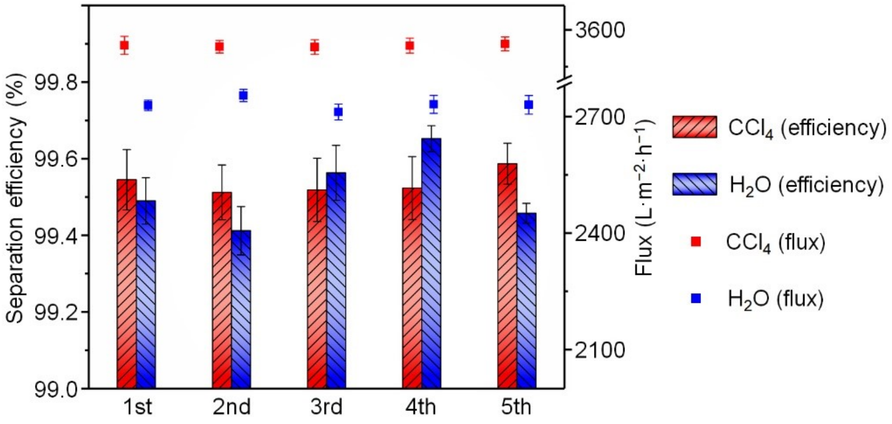

Supplementary Figure 56: The evaluation of the separation efficiency and flux for the membrane after one to five uses in the separation of CCl_4 and H_2O under gravity. Source data are provided as a Source Data file.

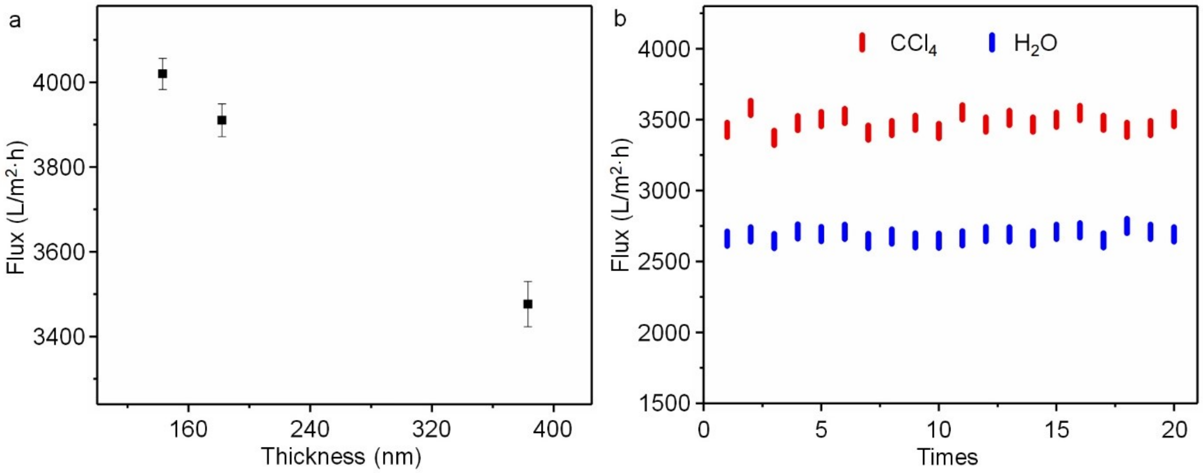

Supplementary Figure 57: (a) The changes flux values of CCl_4 with the increase of the hydrophobic membrane thickness and **(b)** the flux changes of CCl_4 and H_2O for hydrophobic and hydrophilic membranes with thickness of 383 nm versus the consecutive separation up to 20 times. Source data are provided as a Source Data file.

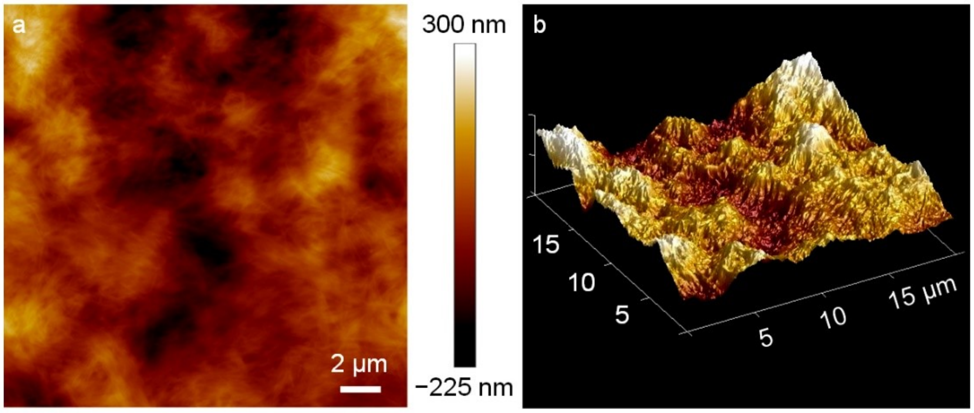

Supplementary Figure 58: Surface roughness of the separation membrane from **(a)** 2D and **(b)** 3D AFM images. Source data are provided as a Source Data file.

Supplementary Discussions

Interfacial energy analysis of separation membrane based on minimization of system's free energy

A reference model [5] shown in Supplementary Figure 59 is used to calculate the total interfacial energy values of the membranes that are completely wetted under different conditions by subjecting to the following assumptions: (1) the infused liquid formed a liquid layer on the surface of the membrane and higher than the surface of the membrane; (2) the thickness of the infused liquid layer is much less than the capillary length of the liquid; (3) surface roughness is uniformly distributed for each configuration; (4) infused liquids are chemically inert with the membrane.

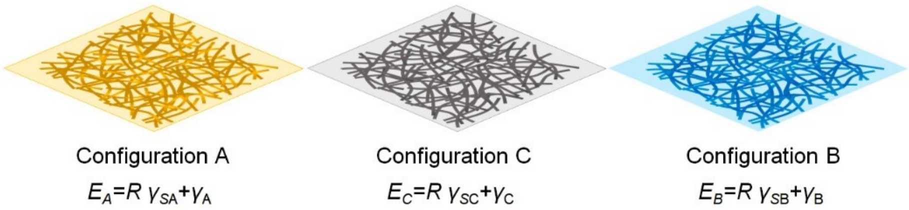

Supplementary Figure 59: Proposed hydrophobic, amphiphilic, and hydrophilic states on separation membrane surface.

At the condition that configuration A is always at a higher energy state than configuration B, there is $\Delta E = E_A - E_B > 0$, which is further expressed as:

$$\Delta E = E_A - E_B = R(\gamma_{SA} - \gamma_{SB}) + \gamma_A - \gamma_B \quad (1)$$

, where γ_A and γ_B represent the surface tension of liquid A and B, γ_{SA} and γ_{SB} represent the surface tension of liquid A and liquid B at solid interface, and R denotes the surface roughness factor (it was fixed at 2). In particular, to be used with measurable quantities, equation (1) that incorporates Young's equation becomes:

$$\Delta E = E_A - E_B = R(\gamma_B cos\theta_B - \gamma_A cos\theta_A) + \gamma_A - \gamma_B \quad (2)$$

, where θ_A and θ_B are the static contact angles of liquid A and liquid B on a flat solid surface, respectively.

On the other hand, according to Laplace theory, the intrusion pressure can be calculated from:[6]

$$\Delta P = \frac{2\gamma cos\theta}{r} = \rho g h \quad (3)$$

, where ΔP is the pressure change, γ represents the surface tension of liquid, θ denotes the contact angle of the liquid on the surface of membrane at equilibrium state, r is the radius of the pores, ρ is the density of the liquid, g (9.81 m·s^{-2}) is the gravitational acceleration speed, and h is the height of the repellent liquid.

In the air state, the membrane is hydrophobic with a water CA of 125±3°. In the data in Supplementary Table 3, the membrane is wettable preferentially by CCl_4 to form stable liquid-infused layer, and becomes hard to be displaced by water. As a result, water will be repelled within the calculated pressure.

In the completely wetting state under liquid which is comparable to the infusion, the measured contact angles are summarized in Supplementary Table 4**.** In this case, the contact angles of miscible liquids should be 0º. The obtained data reveal that under the liquids, the free energy differences of the membrane wetted by miscible liquids are always higher than the one socked with immiscible liquids. As a result, the immiscible liquids are cut off.

Supplementary Table 3: Summary of the data calculated from energetic equations based on contact angles in the air.

Sample state	Liquid A	Liquid B	R	γ_A (mN·m^{-1})	γ_B (mN·m^{-1})	θ_A	θ_B	ΔE (J·m^{-2})	ILL[a]	ΔP (KPa)	h (10^4 m)
In air	CCl_4	H_2O	2	26.8	72.8	4	128	-189.1	CCl_4	341.4	3.5
	H_2O	CCl_4	2	72.8	26.8	128	4	189.1			

a: stable infused-liquid-layer.

Supplementary Table 4: Summary of the data calculated from energetic equations based on contact angles under liquids.

Sample state	Liquid A	Liquid B	R	γ_A (mN·m^{-1})	γ_B (mN·m^{-1})	θ_A	θ_B	ΔE (J·m^{-2})	ILL[a]	ΔP (KPa)	h (10^4 m)
Under CCl_4	CCl_4	H_2O	2	26.8	72.80	0	165	−240.2	CCl_4	534.7	5.5
	H_2O	CCl_4	2	72.8	26.8	165	0	240.2			
Under Tol	Tol	H_2O	2	27.9	72.8	0	156	−231.4	Tol	506.2	5.2
	H_2O	Tol	2	72.8	27.9	150	0	231.4			
Under n-hex	n-hex	H_2O	2	17.9	72.8	0	161	−228.4	n-hex	523.9	5.3
	H_2O	n-hex	2	72.8	17.9	161	0	228.4			
Under H_2O	CCl_4	H_2O	2	26.8	72.8	137	0	138.7	H_2O	94.2	1.0
	H_2O	CCl_4	2	72.8	26.8	0	137	−138.7			
	Tol	H_2O	2	27.9	72.8	139	0	143.2		187.4	1.9
	H_2O	Tol	2	72.8	27.9	0	139	−143.2			
	n-hex	H_2O	2	17.9	72.8	134	0	115.6		138.3	1.4
	H_2O	n-hex	2	72.8	17.9	0	134	−115.6			

a: stable infused-liquid-layer.

Dissipative particle dynamics and simulation model

Dissipative particle dynamic (DPD) simulation, introduced by Hoogerbrugge and Koelman,[7] represents a particle-based method for simulating the dynamic behavior of isothermal liquids on greater length and time scales. Generally, a DPD particle on behalf of a group of atoms or a volume of fluid that is large on atomistic scale but still macroscopically small. This model consists of six kinds of particles, i.e. blue one is water, orange one is carbon tetrachloride, gray one is joystick solvent methanol, green one is POM, yellow one is methyl-pillar[5]arene (for short as "MP"), and pink one is linker (for short as "BCB"). For simplicity, the particles are equal on mass and volume, m is the mass of particles, r_c is the radius of interaction cutoff, and k_BT is the thermal energy, they are set as the units of the simulations, $m = r_c = k_BT = 1$.

The interaction acting on each particle is composed of three parts at least: a conservative force F^C, a dissipative force F^D, and a random force F^R:

$$\mathrm{F}_{ij} = \mathrm{F}_{ij}^C + \mathrm{F}_{ij}^D + F_{ij}^R. \quad (4)$$

They are given by:

$$\mathrm{F}_{ij}^C = \begin{cases} \alpha_{ij}\left(1 - \frac{r_{ij}}{r_c}\right)e_{ij} & r_{ij} < r_c \\ 0 & r_{ij} \geq r_c, \end{cases} \quad (5)$$

$$\mathrm{F}_{ij}^D = -\gamma\omega^D(r_{ij})(\mathrm{v}_{ij} \cdot \mathrm{e}_{ij})\mathrm{e}_{ij}, \quad (6)$$

$$\mathrm{F}_{ij}^R = \sigma\omega^R(r_{ij})\xi_{ij}\Delta t^{-\frac{1}{2}}e_{ij}. \quad (7)$$

Here $\mathbf{r}_{ij} = \mathbf{r}_i - \mathbf{r}_j$, $\mathrm{r}_{ij} = |\mathbf{r}_{ij}|$, $\mathbf{e}_{ij} = \mathbf{r}_{ij}/r_{ij}$, and $\mathbf{v}_{ij} = \mathbf{v}_i - \mathbf{v}_j$. ξ_{ij} are random numbers with zero mean and unit variance. Two weight functions, ω^D and ω^R, are represent dissipative and random forces respectively, that are define according to Español and Warren[8],

$$\omega^D(r_{ij}) = [\omega^R(r_{ij})]^2 \quad , \quad \sigma^2 = 2\gamma k_BT. \quad (8)$$

We choose a simple equation of ω^D and ω^R following Groot and Warren[7]:

$$\omega^D(r_{ij}) = [\omega^R(r_{ij})]^2 = \mathrm{F}_{ij}^C = \begin{cases} \left(1 - \frac{r_{ij}}{r_c}\right)^2 & r_{ij} < r_c \\ 0 & r_{ij} \geq r_c. \end{cases} \quad (9)$$

The friction parameter is $\gamma = 4.5$. The canonical ensemble simulations are performed in a three-dimensional cubic box of size 46^3 with periodic boundaries.[9] Interactions between particles changing with time obeys Newton's motion equations.[10] The particle number density $\rho = 3$, and the step of integration time $\Delta t = 0.02$. The framework model is $[(P_4POM)BCB_2]n$, BCB linked two pillar[5]arenes, in which, the force $F_i^S = \sum_j k r_{ij}$, and $k = 4$.

The interaction parameters α_{ij} , which is a constant which describes the maximum repulsion force between interacting particles, in Eq. (5) are listed in Supplementary Table 5. Definitely, $\alpha_{WW} = \alpha_{GG} = \alpha_{TT} = \alpha_{PP} = \alpha_{MM} = \alpha_{POMPOM} = 25$, which reflects the particles are incompressible at room temperature. Commonly, if α_{ij} is smaller than 25, particles *i* and *j* are "attractive"; otherwise, they are "repulsive". In fact, $\Delta\alpha_{ij} = \alpha_{ij} - 25$ maps to Flory-Huggins χ parameter [11] by the equation: $\Delta\alpha_{ij} = 3.27\chi_{ij}$. For the solvent system, the critical condition corresponds to $\alpha_{ij} = 27.3$[12], in which, $\alpha_{ij} > 27.3$ means they are immiscible or insoluble, while means miscible or soluble.

Supplementary Table 5: The interaction parameters α_{ij} in the simulation model.

	Methanol	Water	CCl_4	POM	MP	BCB
Methanol	25	27	27	23	23	23
Water	27	25	40	23	40	40
CCl_4	27	40	25	40	27	27
POM	23	23	40	25	40	40
MP	23	40	27	40	25	27
BCB	23	40	27	40	27	25

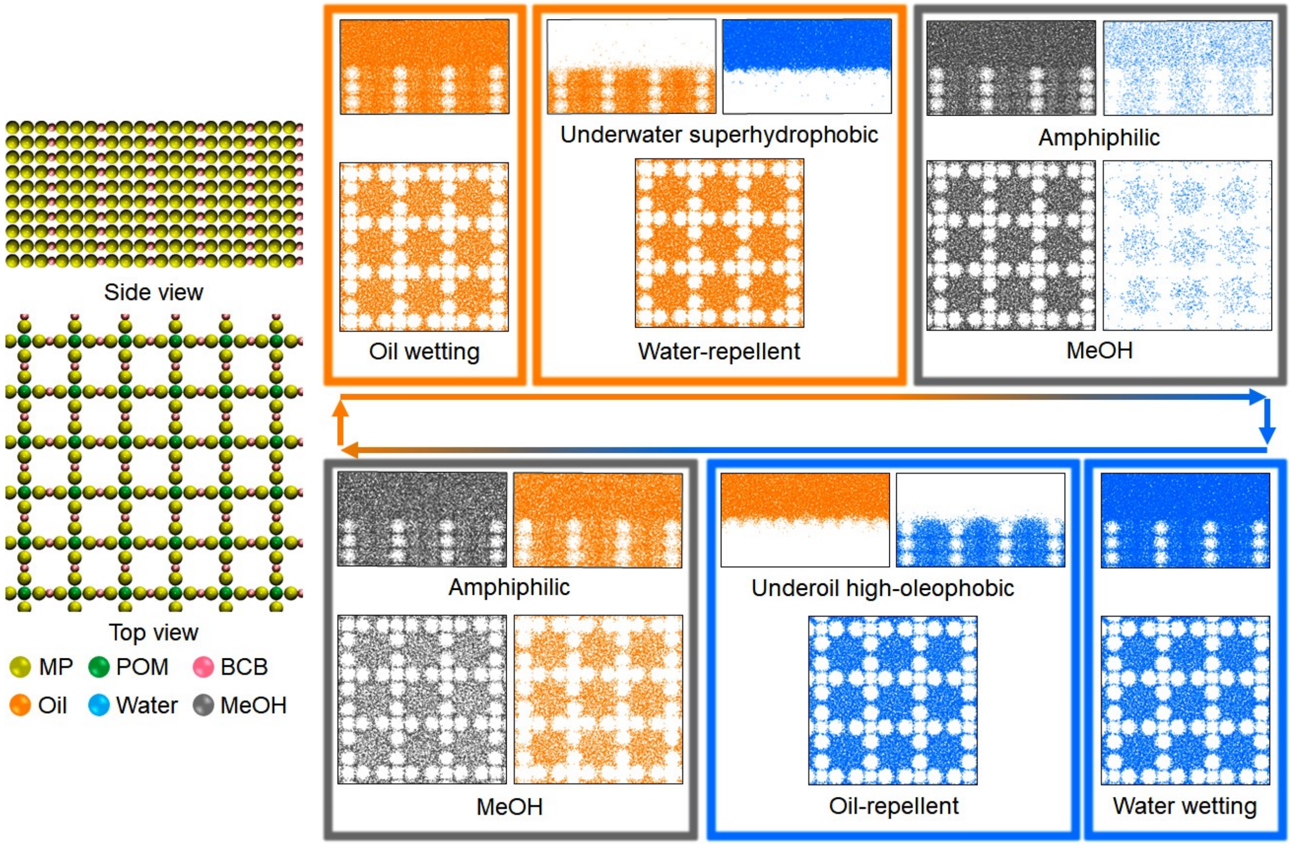

Supplementary Figure 60: Dissipative particle dynamic (DPD) simulation of the liquid separation process. This model consists of six kinds of particles, i.e. blue one is water, orange one is carbon tetrachloride, gray one is joystick solvent methanol, green one is POM, yellow one is methyl-pillar[5]arene (for short as "MP"), and pink one is linker (for short as "BCB"). Source data are provided as a Source Data file.

Supplementary References:

[1] G. A. Tsigdinos, C. J. Hallada, Inorg. Chem. 1968, 7, 437.

[2] P. J. Domaille, J. Am. Chem. Soc. 1984, 106, 7677−7687.

[3] N. L. Strutt, R. S. Forgan, J. M. Spruell, Y. Y. Botros and J. F. Stoddart, J. Am. Chem. Soc. 2011, 133, 5668−5671.

[4] F. Leroy; P. Miró; J. M. Poblet; B. Carles; J. B. Ávalos. J. Phys. Chem. B. 2008, 112, 8591−8599.

[5] T. S. Wong; S. H. Kang; S. K. Tang; E. J. Smythe; B. D. Hatton; A. Grinthal and J. Aizenberg, Nature, 2011, 477, 443−447.

[6] J. P. Youngblood and T. J. McCarthy, Macromolecules, 1999, 32, 6800−6806.

[7] P. J. Hoogerbrugge and J. M. V. A. Koelman, Europhys. Lett. 1992, 19, 155−160.

[8] P. Español and P. Warren, Europhys. Lett. 1995, 30, 191−196.

[9] M. P. Allen a D. J. Tildesley, Computer simulation of liquids. Clarendon Press: Oxford, U.K., 1−382 (1987).

[10] R. D. Groot, P. B. Warren, J. Chem. Phys. 1997, 107, 4423−4435.

[11] R. D. Groot, T. J. Madden, J. Chem. Phys. 1998, 108, 8713−8724.

[12] H. M. Gao, H. Liu, Z. Y. Lu, Z. Y. Sun, L. J. An, J. Chem. Phys. 2013, 138, 224905.
