# Supplementary material for: Processing supramolecular framework for free interconvertible liquid separation
Source: Nat Commun. 2020 Jan 22;11:425. doi: 10.1038/s41467-019-14227-6 (PMC6976700; doi:10.1038/s41467-019-14227-6)
Supplement: Supplementary file 3 — Description of Additional Supplementary Files [file 41467_2019_14227_MOESM3_ESM.pdf]

The movie descripts the in-situ consecutive switchable separation process of water and CCl_4 with the help of the joystick liquid methanol. The oily liquid in red color is CCl_4 (dyed with oil red), the other liquid in blue color is water (dyed with methylene blue), and the colorless liquid is methanol. At the initial state, the membrane is hydrophobic, the mixture of water and CCl_4 is poured onto the membrane, red CCl_4 passes though the membrane, while blue water is blocked. Then, colorless methanol is added to allow water to access the membrane, and after washing with water again, the membrane becomes hydrophilic. At this time, when adding the mixture of water and CCl_4, blue water passes through the membrane, while red CCl_4 is blocked. Further adding methanol to bring CCl_4 through the membrane and then washing with CCl_4 again, the membrane backs to hydrophobic state. Hereto, the switchable separation process accomplishes a conversion cycle.
